# MISP regulates the IQGAP1/Cdc42 complex to collectively orchestrate spindle orientation and mitotic progression

**DOI:** 10.1038/s41598-018-24682-8

**Published:** 2018-04-20

**Authors:** Barbara Vodicska, Berati Cerikan, Elmar Schiebel, Ingrid Hoffmann

**Affiliations:** 1 Cell Cycle Control and Carcinogenesis, F045, German Cancer Research Center, DKFZ, 69120 Heidelberg, Germany; 2 Zentrum für Molekulare Biologie der Universität Heidelberg (ZMBH), DKFZ – ZMBH Alliance, Im Neuenheimer Feld 282, 69120 Heidelberg, Germany; 3 Heidelberg University, Heidelberg, Germany

## Abstract

Precise mitotic spindle orientation is essential for both cell fate and tissue organization while defects in this process are associated with tumorigenesis and other diseases. In most animal cell types, the dynein motor complex is anchored at the cell cortex and exerts pulling forces on astral microtubules to position the spindle. The actin-binding protein MISP controls spindle orientation and mitotic progression in human cells. However, the exact underlying mechanism remains to be elucidated. Here we report that MISP interacts with the multidomain scaffolding protein IQGAP1. We further show that MISP binds to the active form of Cdc42 through IQGAP1. Depletion of MISP promotes increased accumulation of IQGAP1 at the cell cortex and a decrease in its Cdc42-binding capacity leading to reduced active Cdc42 levels. Interestingly, overexpression of IQGAP1 can rescue mitotic defects caused by MISP downregulation including spindle misorientation, loss of astral microtubules and prolonged mitosis and also restores active Cdc42 levels. Importantly, we find that IQGAP1 acts downsteam of MISP in regulating astral microtubule dynamics and the localization of the dynactin subunit p150^glued^ that is crucial for proper spindle positioning. We propose that MISP regulates IQGAP1 and Cdc42 to ensure proper mitotic progression and correct spindle orientation.

## Introduction

Orientation of the mitotic spindle is a fundamental process in all multicellular organisms crucial for both symmetrical and asymmetrical cell divisions. Failure of correct division orientation may lead to developmental disorders and cause tumor formation. External cues, which are thought to be transmitted via cortical signals, also contribute to this complex mechanism to determine the correct division axis in mitotic cells^1^. The actin-binding protein MISP (mitotic interactor and substrate of Plk1) functions in spindle orientation probably by linking the astral microtubules (MTs) to the actin cytoskeleton^2–4^. Lack of MISP leads to mitotic defects including spindle orientation and positioning disorders, loss of astral microtubules, prolonged metaphases accompanied by SAC activation, chromosome misalignment, disrupted poles and increased mitotic index^2^.

IQGAP1, an evolutionarily conserved 190-kDa scaffolding protein that mediates the formation of various protein complexes, is required for many cellular processes including spindle orientation. IQGAP1 was shown to be involved in regulating spindle orientation through restricting the distribution of NuMA to the basolateral membrane^5^ and it also regulates the capture and stabilization of astral MTs at the cell cortex in collaboration with its binding partner CLIP-170^6,7^. One of the best-characterized interaction partners of IQGAP1 is the small GTPase Cdc42. Together they regulate crosslinking of actin filaments, MT dynamics and E-cadherin-mediated cell–cell adhesion^8^. Despite its name and the domain homology, IQGAP1 is not a GTPase accelerating protein (GAP). Due to a point mutation in the active site of the GAP domain, IQGAP1 cannot hydrolyze GTP but instead, it has been reported to stabilize GTP-bound active Cdc42 and Rac1. Therefore overexpression of IQGAP1 causes an increase in the levels of GTP-bound Cdc42^9,10^. Notably, Cdc42 also plays a role in spindle orientation, since knockdown of Cdc42 leads to misoriented spindles^11,12^.

IQGAP1 seems to play an important role in tumorigenesis and metastasis. IQGAP1 was found to be overexpressed in a number of human tumors^13^, and increased IQGAP1 staining can be observed at cell-cell contacts at the invasive front of certain tumour samples^14^. Moreover, xenograph experiments showed that IQGAP1 enhances mammary tumorigenesis^15^.

In this report we show that MISP directly interacts with IQGAP1 and regulates its subcellular localization. MISP depletion leads to accumulation of IQGAP1 at the cell cortex in mitotic cells. Upon MISP downregulation, IQGAP1 fails to stabilize active Cdc42 which in turn leads to a decrease in active Cdc42 levels. We further show that overexpresion of IQGAP1 can rescue mitotic defects including spindle misorientation, loss of astral microtubules and prolonged mitosis caused by MISP downregulation while it also restores active Cdc42 levels. Our results suggest that MISP collaborates with IQGAP1 and Cdc42 to collectively orchestrate spindle orientation and mitotic progression.

## Results

### MISP interacts and co-localizes with IQGAP1

**Figure 1:**
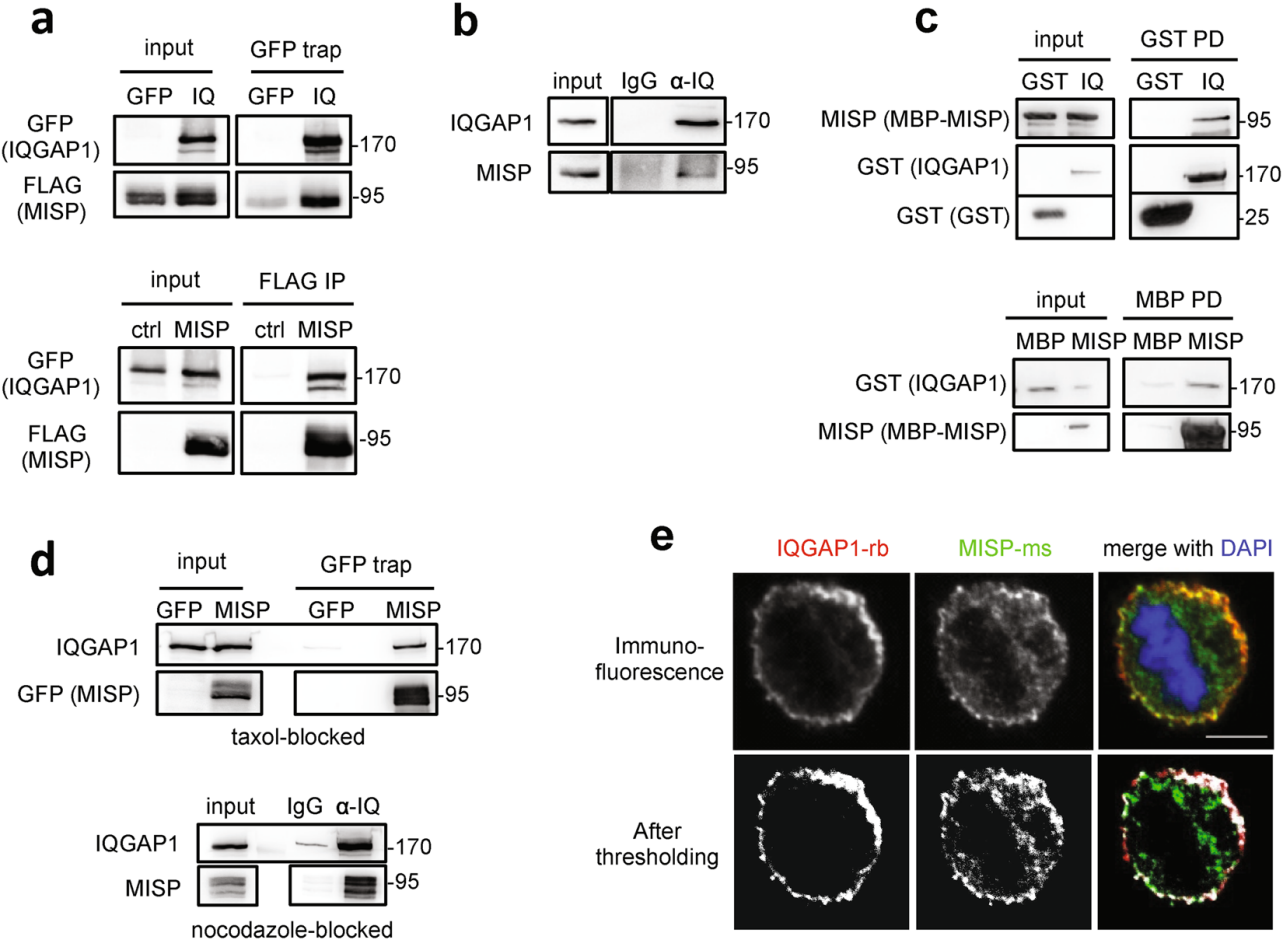
MISP interacts and co-localizes with IQGAP1. (**a**) HEK293T cells transiently overexpressing GFP-IQGAP1 and FLAG-MISP were used for co-immunoprecipitation experiments from both sides (GFP trap or FLAG M2 beads). (**b**) Endogenous immunoprecipitation using IQGAP1 antibody or control immunoglobulin (IgG) was carried out in HeLa cell lysates and MISP was detected in the eluate. (**c**) *In vitro* interaction between purified MBP-MISP and GST-IQGAP1 was detected by GST and MBP pull-down experiments. (**d**) Immunoprecipiation experiments showing that GFP-MISP binds endogenous IQGAP1 (upper panel) and MISP binds to IQGAP1 endogenously using specific antibodies (lower panel) in mitotically arrested (taxol and nocodazole, respectively) HeLa cell lysates. (**a–d**) Images were gained from the same Western blot membrane after cutting/cropping and presented with different exposure times or contrast enhancement for better presentation purposes. The dividing lane marks the grouping of images of the same (or different) membrane. Uncropped blots, where applicable, are included in Supplementary Fig. 4. (**e**) HeLa cells were immunostained for MISP and IQGAP1 and co-localization was visualized in mitosis in single-plane confocal images, scale bar: 5 μm. Lower pictures: After manual thresholding white spots mark the co-localizing areas in the merge images.

To better understand the mechanisms underlying the function of MISP in spindle orientation and mitotic progression, biochemical screens were performed to identify novel MISP interaction partners. Using co-immunoprecipitation (co-IP) followed by mass spectrometry analysis with MISP as bait in mitotically blocked HeLa cells we identified the scaffolding protein IQGAP1 (Supplementary Fig. 1a), which has already been indicated in a previous screen as a potential interaction partner of MISP^16^ and which was shown to be involved in spindle orientation^5^. The interaction between the two proteins was confirmed by co-IP experiments with overexpressed proteins in asynchronous HEK 293 T cell lysates (Fig. 1a). In addition, MISP and IQGAP1 form an *in vivo* complex as endogenous MISP binds endogenous IQGAP1 (Fig. 1b). Interaction studies with the N- and C-terminal half of IQGAP1 revealed that MISP strongly binds to the C-terminal part of IQGAP1 (Supplementary Fig. 1b,c). Interestingly, the phosphorylation status of MISP by Plk1 did not show an effect on its binding to IQGAP1, IQGAP1 co-precipitated with both the phospho-mimicking (6DP) and the phospho-deficient (7AP) mutants of MISP to a comparable extent (Supplementary Fig. 1d). Next we asked whether the interaction between IQGAP1 and MISP was direct. *In vitro* pull-down assays using recombinant proteins confirmed that IQGAP1 and MISP also form a complex *in vitro* (Fig. 1c). Furthermore, co-immunoprecipiation experiments in HeLa cells showed that the IQGAP1-MISP interaction is also present in cells that were arrested in mitosis (Fig. 1d). The interaction between the two proteins was also supported by their co-localization at cortical regions in mitotic HeLa cells (Fig. 1e).

### MISP regulates IQGAP1 distribution at the cell cortex in mitosis in a Cdc42-dependent manner

**Figure 2:**
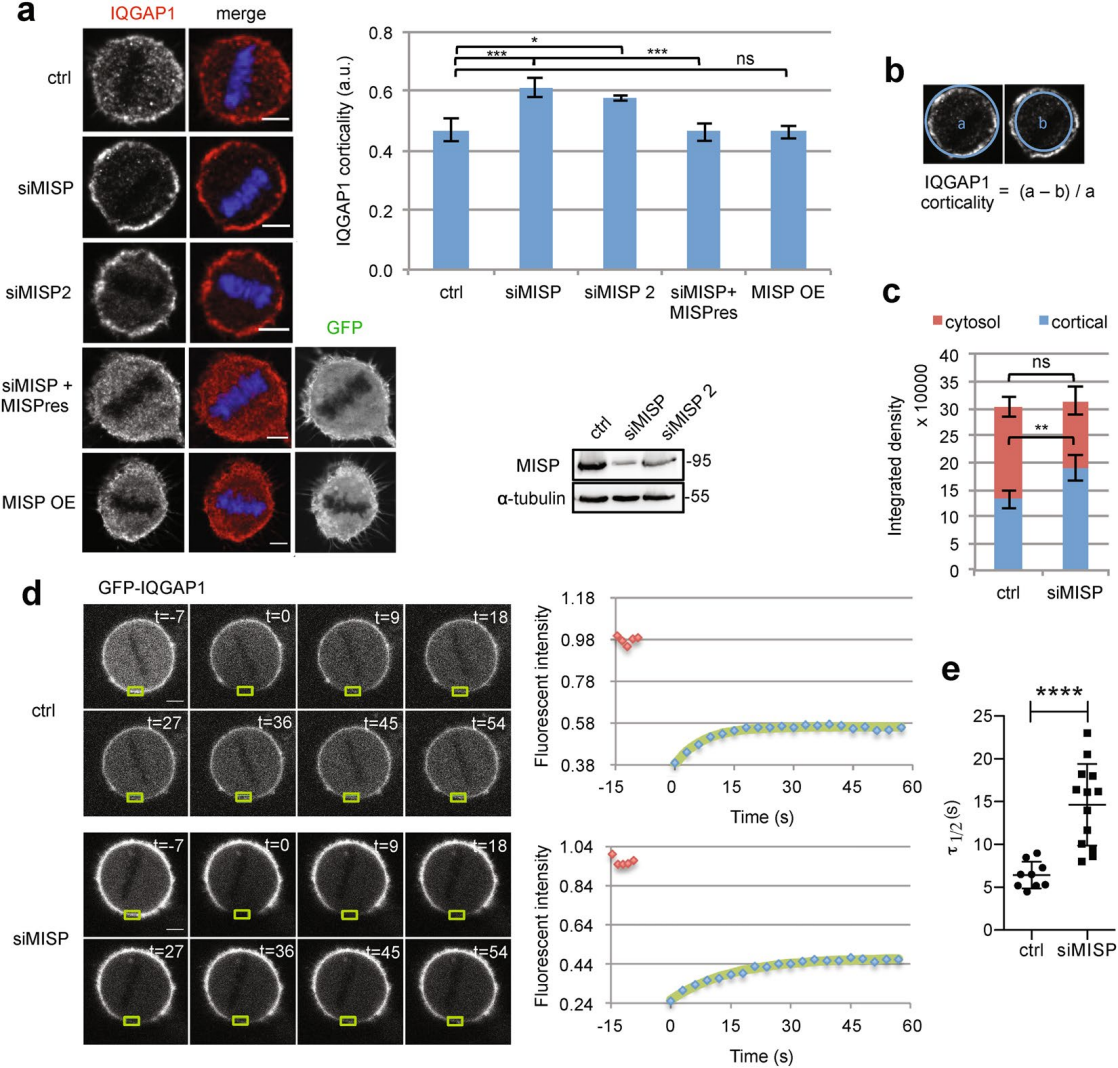
Loss of MISP leads to cortical accumulation of IQGAP1 in mitotic cells. (**a**) Left: Mitotic HeLa Kyoto cells treated with control or either MISP siRNA or a GFP-MISP construct were immunostained for IQGAP1. MISPres, siRNA-resistant form of MISP. Single equatorial images, scale bar: 5 μm. Chart: Corresponding quantification of IQGAP1 signal at the cortex in control, MISP KD, rescue or MISP OE conditions. Values represent mean ± SD of 3 independent experiments, n = 15, one-way ANOVA with Bonferroni’s test. WB, Immunoblot showing downregulation efficiency of MISP with two different siRNAs. Images were gained from the same Western blot membrane. (**b**) Illustration of how the cortical IQGAP1 signal was quantified. (**c**) Quantification of the distribution of IQGAP1 between cortex and cytosol in control and MISP-depleted mitotic HeLa Kyoto cells, n = 15, p < 0.01. (**d**) FRAP experiments of control and MISP siRNA-treated HeLa cells inducibly overexpressing GFP-IQGAP1. Green rectangles mark the bleached areas, scale bar: 5 μm. Red squares on the graphs indicate pre-bleaching data, blue squares show recovery after bleaching with a green single exponential trend line. (**e**)**τ**_1/2_ of FRAP experiments shows recovery half-time of control and MISP-depleted cells in one representative experiment, p < 0.0001.

To gain insight into the functional consequences of the interaction between MISP and IQGAP1, we analyzed the distribution of IQGAP1 at the cortex upon MISP downregulation. We found that IQGAP1 localized both in the cytosol and at the cell cortex in mitotic cells (Fig. 2a). Interestingly, siRNA-mediated depletion of MISP increased the cortical localization of IQGAP1 in three different human cell lines (Fig. 2a,b, Supplementary Fig. 2a). The phosphorylation status of MISP by Plk1 did not seem to influence the cortical accumulation of IQGAP1, since both siRNA-resistant mutants (6DP and 7AP) could rescue the cortical accumulation of IQGAP1 upon depletion of the endogenous protein (Supplementary Fig. 2b). The cortical elevation of IQGAP1 could be rescued upon expression of a siRNA-resistant version of MISP (Fig. 2a). In contrast, IQGAP1 depletion did not have an effect on the cortical localization of MISP in mitosis (Supplementary Fig. 2c). We also did not observe an effect on IQGAP1 levels at the cell cortex upon ectopic expression of MISP (MISP OE) (Fig. 2a). While upon MISP downregulation cortical levels of IQGAP1 increased, cytosolic IQGAP1 levels decreased and the overall intensity of IQGAP1 within the cells did not change (Fig. 2c) suggesting that IQGAP1 might be recruited from the cytosol to the cell cortex. This is in line with the observation that the cellular levels of IQGAP1 did not change after MISP knock-down (KD) (Supplementary Fig. 2a). Correspondingly, using fluorescence recovery after photobleaching (FRAP) we observed a slower recovery of GFP-IQGAP1 after MISP depletion at cortical regions of mitotic cells than after control siRNA treatment (Fig. 2d,e), which could arise from the decreased amount of cytosolic fraction. The immobilized fraction was not affected (Supplementary Fig. 2d), suggesting that IQGAP1 might not get immobilized near the plasma membrane via a strong interaction.

**Figure 3:**
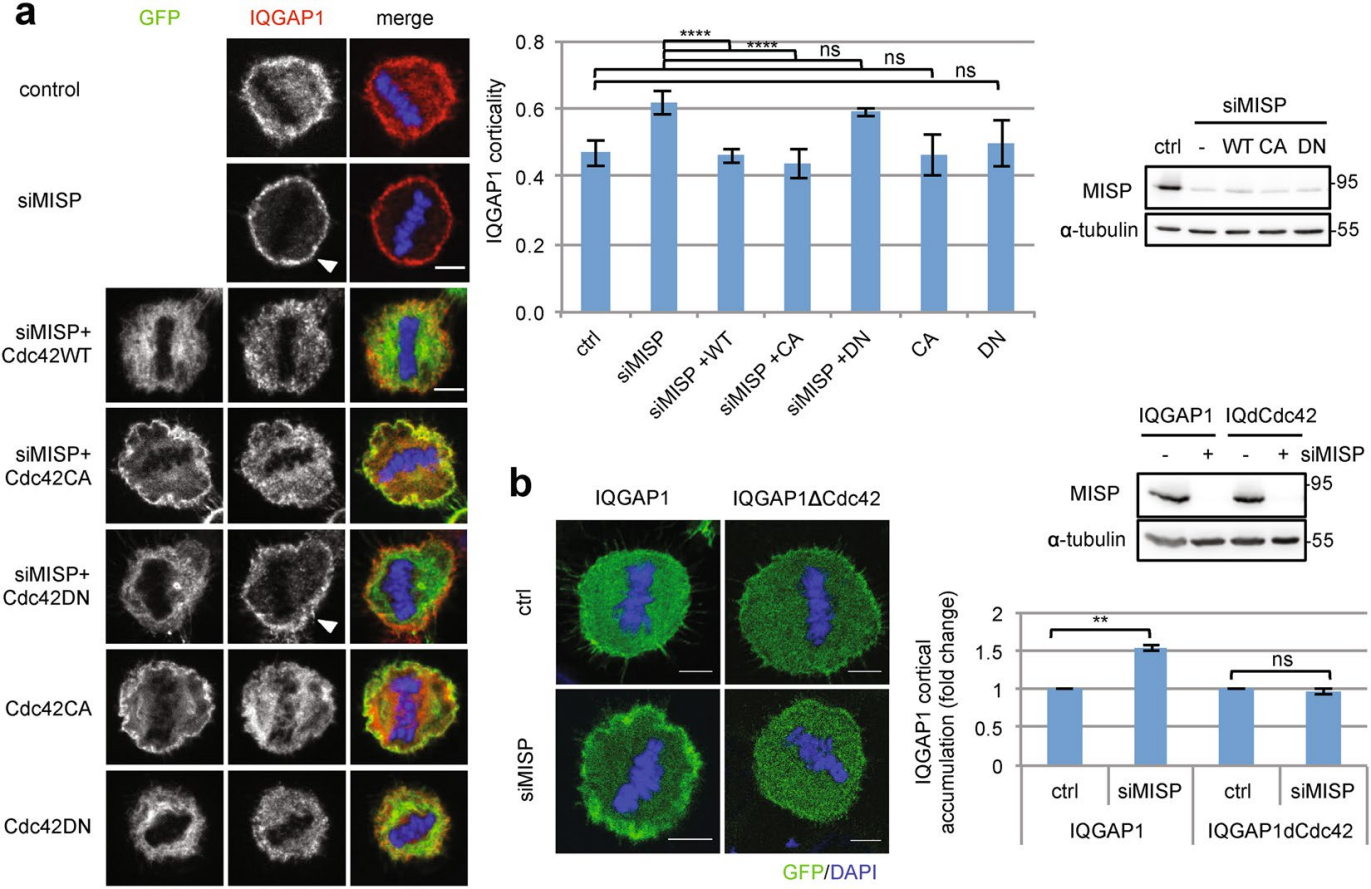
MISP controls cortical accumulation of IQGAP1 in a Cdc42-dependent manner. (**a**) Immunostaining for IQGAP1 in control and MISP-depleted mitotic HeLa Kyoto cells overexpressing different Cdc42 constructs. Arrowheads show cortically accentuated IQGAP1. Single equatorial images, scale bar: 5 μm. Chart: IQGAP1 corticality was quantified as in Fig. 2b. Values represent mean ± SD of 3 independent experiments, n = 15, one-way ANOVA with Bonferroni’s test. WB: Immunoblotting showing MISP down-regulation in different conditions. (**b**) Cortical accumulation of overexpressed WT or Cdc42-binding-deficient IQGAP1 (IQdCdc, Δaa1054-77) was analyzed in control and MISP siRNA treated HeLa Kyoto cells in mitosis by fluorescence microscopy. Chart: Cortical accumulation of the constructs was measured in control and siMISP cells like in Fig. 2b. Results were normalized to control and one-sample t-test was carried out on log_2_-transformed data. Values represent mean ± SD of 3 independent experiments, p = 0.0017 and 0.4104. Western blot shows MISP downregulation using siRNAs. (**a-b**) Blot images were gained from the same Western blot membrane.

We found that Akt, another downstream partner of IQGAP1 also seems to be affected by MISP downregulation, since MISP-depleted cells showed reduced Akt activation (Supplementary Fig. 2e) similar to IQGAP1-depleted cells^13^. The small GTPase family member Cdc42 was shown to be a regulator of IQGAP1 activity and its affinity towards interaction partners^17^. For example, Cdc42 can inhibit the IQGAP1-β-catenin interaction and thereby regulate the subcellular localization of IQGAP1^18^. Furthermore, Cdc42 is also involved in the regulation of spindle orientation^12^. In addition, Cdc42 was found in our screen as a MISP-interacting protein (Supplementary Fig. 1a). Intrigued by these findings we aimed to further address the functional interplay between MISP, IQGAP1 and Cdc42 in spindle orientation. First, we investigated whether overexpression of Cdc42 could regulate IQGAP1 activity by rescuing the cortical accumulation of IQGAP1 upon MISP depletion. To our surprise, expression of both Cdc42 wild-type (WT) and the constitutively active form of Cdc42 (CA, Q61L) could diminish cortical accumulation of IQGAP1 in the absence of MISP, while expression of Cdc42 dominant negative (DN, T17N) did not alter the subcellular distribution of IQGAP1 (Fig. 3a). Importantly, overexpression of the DN form of Cdc42 alone did not trigger the cortical accumulation of IQGAP1 and the CA form did not reduce it, suggesting that the phenotype is specific to MISP and not only caused by the activation status of Cdc42.

IQGAP1 binds to Cdc42 via a short amino acid motif (aa 1054–1077) within the GRD domain^19^. To confirm that the interaction between IQGAP1 and Cdc42 is required for the regulation of IQGAP1 by MISP at the cell cortex we generated a deletion mutant of IQGAP1 that is unable to bind Cdc42 (IQGAP1∆Cdc42, ∆1054-77)^19^. We verified that this mutant cannot bind Cdc42 but still capable of binding MISP (Supplementary Fig. 2f). Unlike IQGAP1WT, IQGAP1∆Cdc42 did not accumulate at the cell cortex in mitosis upon MISP depletion (Fig. 3b). From these data, we conclude that MISP regulates IQGAP1 levels at the cell cortex and that accumulation of IQGAP1 at the cell cortex is dependent on Cdc42.

### MISP interacts with the active form of Cdc42 through IQGAP1

**Figure 4:**
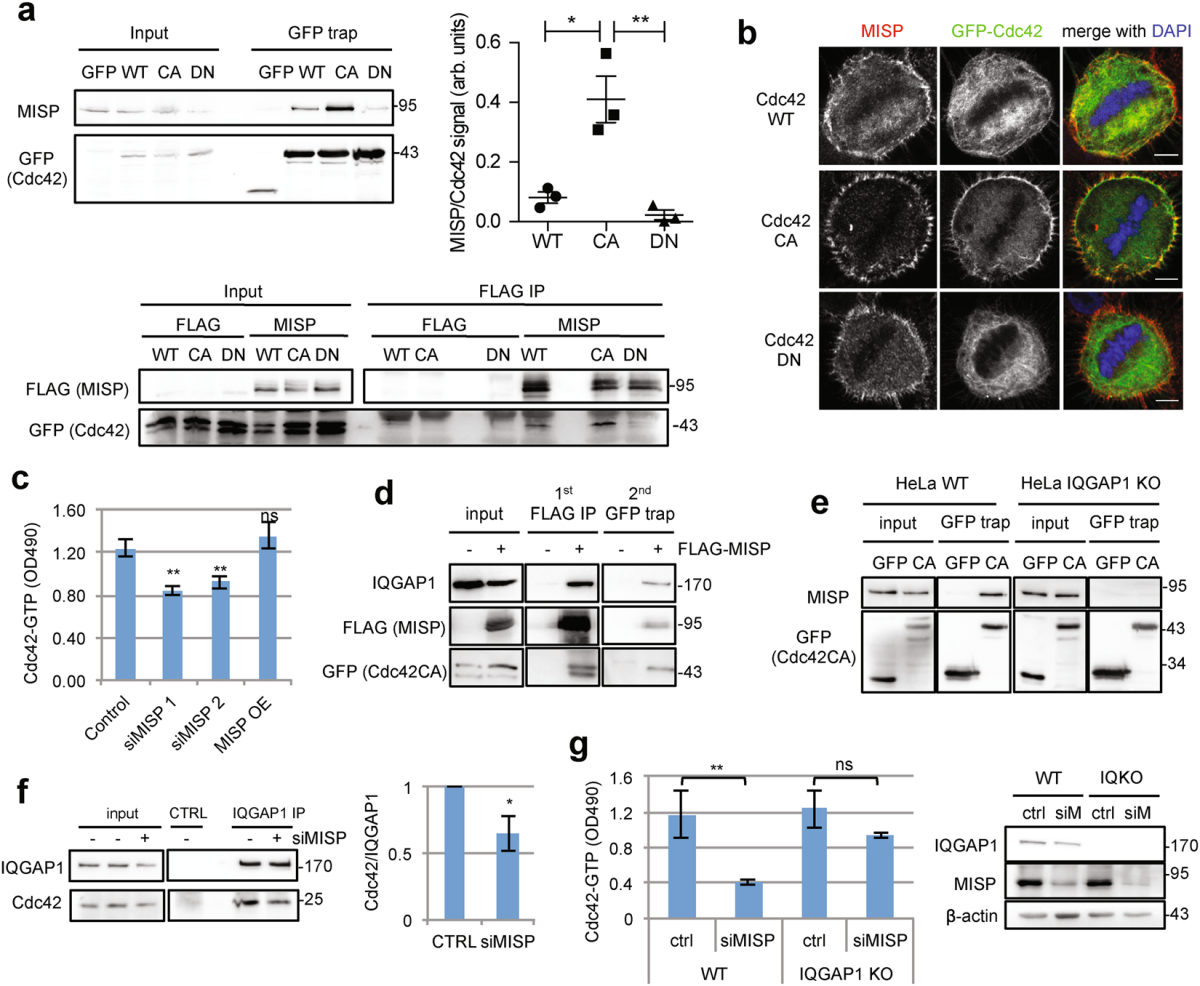
MISP regulates the activity of Cdc42 through IQGAP1. (**a**) Upper blot, representative co-immunoprecipitation experiment of overexpressed Cdc42 mutants and endogenous MISP in HeLa Kyoto cells. Chart, quantification of MISP co-precipitation relative to the amount of Cdc42. Values represent mean ± SD of 3 independent experiments, p = 0.0149, 0.0083. Lower blot: HEK293T cells were transfected with empty FLAG or FLAG-MISP plasmid and GFP-tagged Cdc42 mutants. Following FLAG IP, GFP-Cdc42 co-precipitation was detected by Western blotting. (**b**) MISP immunostaining of HeLa Kyoto cells overexpressing GFP-Cdc42 mutants. Single equatorial plane, scale bar: 5 μm. (**c**) HeLa Kyoto cells were transfected with control/MISP siRNAs or a GFP-MISP plasmid. Active Cdc42 levels were quantified using the Rho G-LISA activation assay (Cytoskeleton). Values represent mean ± SD of 3 independent experiments, one-way ANOVA with Bonferroni’s. (**d**) Sequential immunoprecipitation of HEK293T cells transfected with FLAG-tag or FLAG-MISP and GFP-Cdc42CA to analyze complex formation. First, FLAG-MISP was enriched on beads and then bound proteins were eluted and subjected to a GFP trap to enrich for Cdc42CA. After the final elution IQGAP1 co-precipitation was detected with WB. (**e**) Co-precipitation of endogenous MISP with GFP-Cdc42CA was analyzed in wild-type (WT) and IQGAP1 knock-out (IQGAP1 KO) HeLa cells. (**f**) Endogenous IQGAP1 was immunoprecipitated from control or MISP siRNA-treated HeLa cell lysates. Co-precipitating Cdc42 was quantified relative to bound IQGAP1. Results were normalized to control and one-sample t-test was carried out on log_2_-transformed data. Values represent mean ± SD of 3 independent experiments, p = 0.0471. (**g**) Cdc42 activation experiment of HeLa WT and IQGAP1 knock-out (KO) cells transfected with control or MISP siRNAs (Rho G-LISA activation assay, Cytoskeleton). Values represent mean ± SD of 3 independent experiments, p = 0.0083 and 0.0741. Immunoblot shows downregulation efficiency of MISP. (**a-g**) Blot images were gained from the same Western blot membrane after cutting/cropping and presented with different exposure times for better presentation purposes. The dividing lane marks the grouping of images of the same (or different) membrane. Uncropped blots, where applicable, are included in Supplementary Fig. 4.

Given that Cdc42 regulates the cortical accumulation of IQGAP1 in a MISP-dependent manner we aimed at characterizing the interaction between MISP and Cdc42 in more detail. Co-IP experiments revealed that MISP strongly interacts with the constitutively active form of Cdc42, while only a slight interaction with the wild-type and almost none with the dominant-negative form was detectable (Fig. 4a). We further found that the interaction between MISP and Cdc42CA was also present in mitotic cells (Supplementary Fig. 3a). In addition, MISP also co-localized with Cdc42WT and Cdc42CA but not with Cdc42DN at the cell cortex (Fig. 4b).

Proteins specifically interacting with the active form of GTPases might influence their activation. To find out if MISP could affect the activity of Cdc42, we depleted or overexpressed MISP in HeLa cells and analyzed the activity status of the three most abundant Rho GTPase family members: RhoA, Rac1 and Cdc42. Using a Rho G-LISA activation assay we identified a specific decrease in the level of GTP-bound Cdc42 in response to siRNA-mediated MISP depletion (Fig. 4c). GTP-bound RhoA and Rac1 levels were not affected (Supplementary Fig. 3b). Since downregulation of MISP leads to deactivation of Cdc42 we investigated whether artificial increase in active Cdc42, namely co-expression of a constitutively active Cdc42, could rescue the spindle misorientation effect induced by downregulation of MISP. However, Cdc42CA overexpression did not rescue the spindle misorientation upon MISP knock-down (Supplementary Fig. 3c) as it also did not compensate for the loss of astral MTs in response to MISP KD (Supplementary Fig. 3d). Notably, Cdc42CA OE alone resulted in spindle misorientation and loss of astral MTs, implicating that similarly to deregulation, artificial over-stabilization of active Cdc42 can lead to defects in spindle orientation and astral MT anchoring at the cortex (Supplementary Fig. 3c,d). Importantly, although MISP KD induces Cdc42 deactivation, we could not show that purified MISP would activate Cdc42 by helping it to exchange GDP to GTP (Supplementary Fig. 3e), which is in line with the observation that MISP OE does not lead to an increase in active Cdc42 in the cells (Fig. 4c). These results suggest that MISP itself does not act as an activator, a guanine nucleotide exchange factor (GEF) for Cdc42.

The preferential binding of MISP to active Cdc42 resembles that of IQGAP1 and Cdc42. IQGAP1 was shown to bind and stabilize Cdc42 in its active form^9^, hence it is possible that the three proteins interact in a complex. To this end, we conducted a double sequential immunoprecipitation experiment. FLAG-MISP and GFP-Cdc42CA were co-expressed in HEK 293 T cells and FLAG-MISP was immunoprecipitated followed by GFP trap to enrich for Cdc42CA. Western blot analysis of the eluate revealed that endogenous IQGAP1 specifically co-precipitated with MISP and Cdc42CA (Fig. 4d) confirming the ternary complex formation. In this complex it would be conceivable that MISP interacts with and regulates the activity of Cdc42 through IQGAP1. While the interaction between IQGAP1 and MISP appeared to be direct (Fig. 1c), no specific interaction could be detected between MISP and Cdc42 *in vitro* (Supplementary Fig. 3f), suggesting that MISP binds Cdc42 through IQGAP1. *In vivo* data also supports this hypothesis, since no interaction between MISP and Cdc42CA was detected in an IQGAP1 knock-out (KO) HeLa cell line^20^ (Fig. 4e).

In order to prove that IQGAP1 is indeed the mediator responsible for the decrease in active Cdc42 levels, we performed immunoprecipitation experiments with an IQGAP1 antibody in control and MISP siRNA-treated HeLa cells and measured co-precipitating Cdc42 relative to IQGAP1. There was a clear reduction of Cdc42 bound to IQGAP1 in the absence of MISP, indicating a change in the Cdc42-binding affinity of IQGAP1 (Fig. 4f). Since IQGAP1 binds predominantly to the active form of Cdc42, this could lead to a reduction in overall active Cdc42 levels in the cells that was observed upon MISP downregulation. We strengthened this finding by checking the activation of Cdc42 in wild-type (WT) and IQGAP1 KO HeLa cells and found that unlike in WT cells, MISP depletion did not lead to a deactivation of Cdc42 in IQGAP1 KO cells compared to control siRNA treatment (Fig. 4g). This result supports the hypothesis that IQGAP1 is the mediator in regulating Cdc42 activity downstream of MISP, since in cells lacking IQGAP1, MISP has no effect on the levels of active Cdc42. Collectively, these data indicate that MISP interacts with and regulates the activity of Cdc42 through IQGAP1 binding.

### IQGAP1 acts downstream of MISP in regulating spindle orientation and mitotic progression

**Figure 5:**
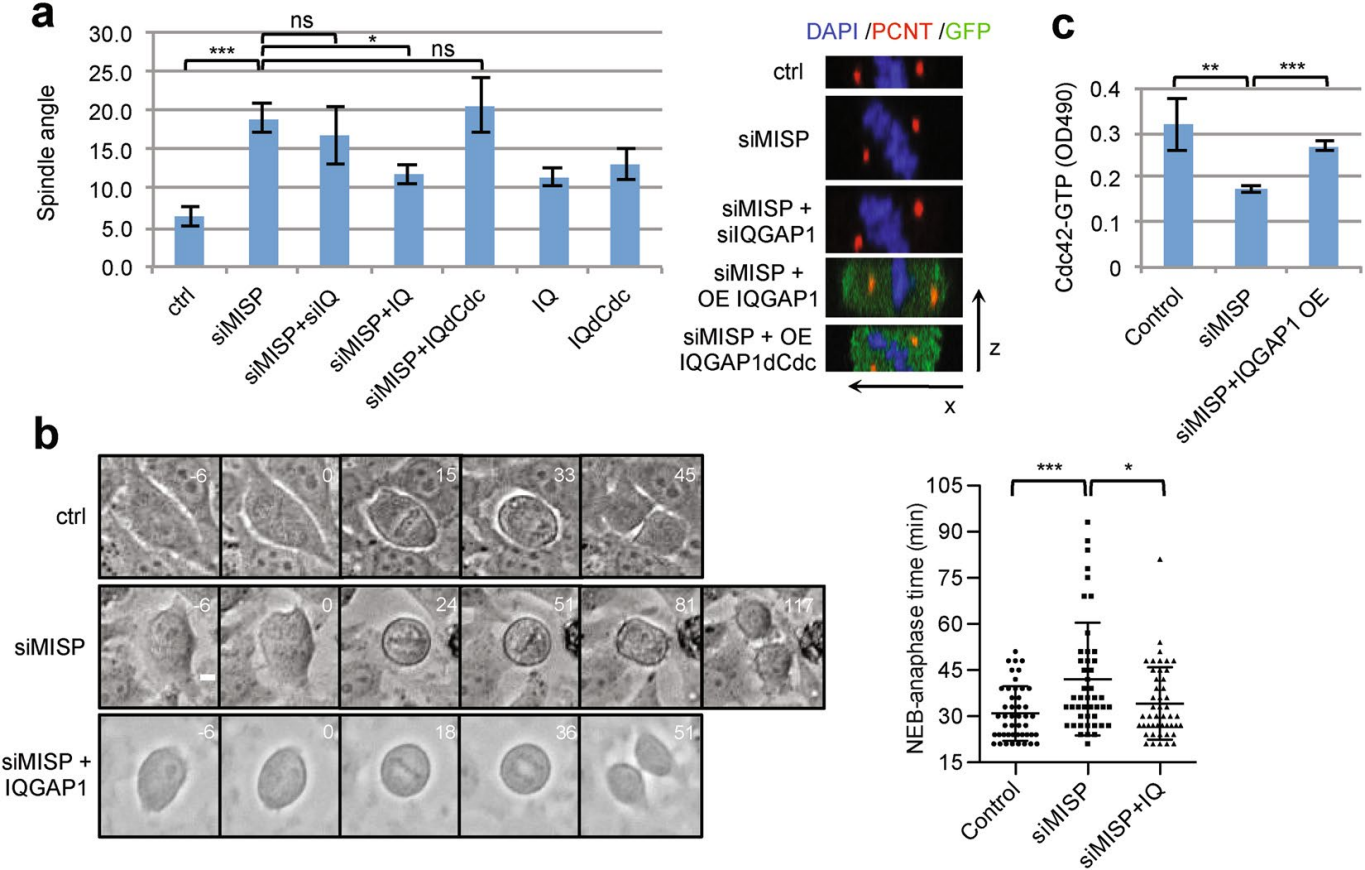
IQGAP1 OE rescues MISP KD phenotypes and restores active Cdc42 levels. (**a**) Spindle angle was measured relative to the substratum in mitotic HeLa Kyoto cells treated with control/MISP/IQGAP1 siRNA and GFP-IQGAP1/GFP-IQGAP1dCdc42 constructs. Values represent mean ± SD of 3 independent experiments, n = 15, p = 0.0007 compared to controls, one-way ANOVA with Bonferroni’s test. Right: Spindle angles are depicted by representative x-z side views, centrosomes were visualized by pericentrin (PCNT) staining. (**b**) NEB-anaphase time of HeLa cells inducibly overexpressing GFP-IQGAP1 was measured by live-cell imaging in control and MISP KD cells. Brightfield images show mitotic progression. Numbers indicate minutes, zero is set to NEB. The next image shows the first congressed metaphase plate (for siMISP also a second metaphase plate was imaged), followed by the onset of mitosis and the settled daughter cells. Scale bar: 10 μm. Chart: Dot plot showing time spent in mitosis. Values represent mean ± SD, n = 46, Mann-Whitney test, p = 0.0007 and 0.0226. (**c**) Active Cdc42 levels were measured in HeLa cell lysates upon IQGAP1 OE after MISP KD (RhoA/Rac1/Cdc42 GLISA, Cytoskeleton). Values represent mean ± SD of 3 independent experiments, p = 0.0072 and 0.0003.

Loss of MISP induces mitotic defects including spindle misorientation accompanied by shortened astral MTs and prolonged mitosis^21^. Trying to eliminate the aberrant cortical accumulation of IQGAP1 by co-depletion of MISP and IQGAP1 did not rescue the spindle misorientation phenotype, suggesting that the cortical accumulation of IQGAP1 is not a cause for spindle orientation defects. Instead, overexpression of IQGAP1 normalized the spindle angles after MISP KD (Fig. 5a). Another phenotype of MISP depletion is the impairment of the metaphase-to-anaphase transition^2^. To find out whether IQGAP1 overexpression could also rescue the metaphase arrest we performed time-lapse video microscopy after MISP depletion in HeLa cells inducibly overexpressing IQGAP1. As described previously^2^, MISP depletion increased the time from nuclear envelope breakdown to anaphase onset from around 30 minutes in control siRNA-treated cells to 45 minutes in MISP-depleted cells, while overexpression of IQGAP1 could reduce this time to 35 minutes in the absence of MISP (Fig. 5b and Supplementary Movies 1–3). Given that MISP depletion causes a decrease in Cdc42 activation (Fig. 4c), we wondered whether the rescue effect of IQGAP1 is attributable to its active Cdc42-stabilizing ability. Indeed, overexpression of IQGAP1 in MISP KD cells could restore the Cdc42-GTP signal to nearly control levels (Fig. 5c). Importantly, expression of IQGAP1∆Cdc42 did not rescue either the spindle misorientation phenotype after MISP KD (Fig. 5a) pointing to the importance of Cdc42-binding of IQGAP1 in the rescue function. Taken together, these results suggest that IQGAP1 overexpression can rescue MISP depletion phenotypes most probably by restoring the levels of active Cdc42 in the cells.

### IQGAP1 compensates for MISP to stabilize astral MTs

**Figure 6:**
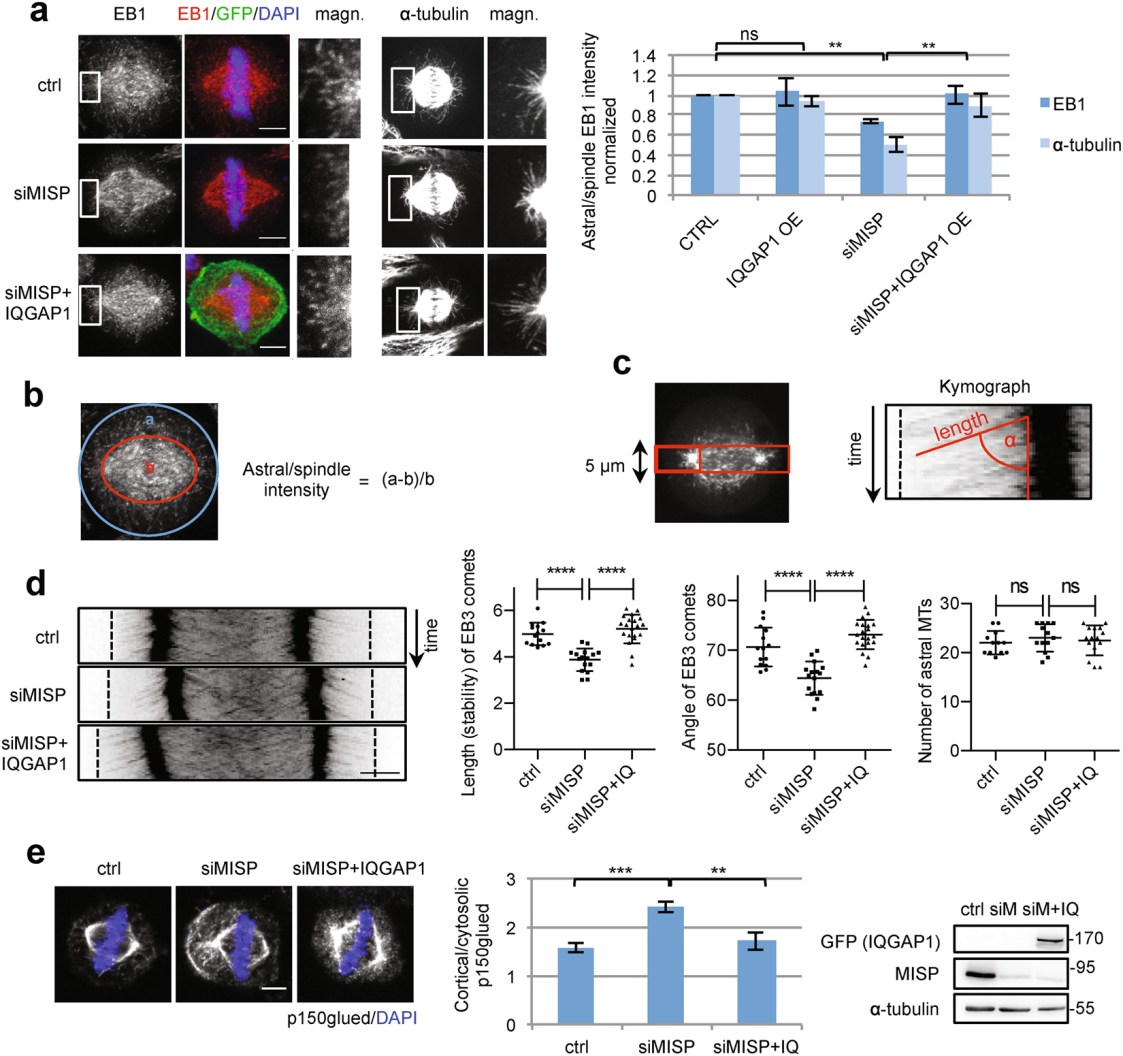
IQGAP1 OE can abrogate the destabilization effect of MISP KD on astral MTs. (**a**) Maximum projections of confocal images showing MT plus ends stained by EB1 or astral MTs stained by α-tubulin in mitotic HeLa cells inducibly overexpressing GFP-IQGAP1 treated with control or MISP siRNA. Scale bar: 5 μm. Rectangles mark magnified areas. Chart: Quantification was carried out in sum projections using ImageJ. Results were normalized to control and one-sample t-test was carried out on log_2_-transformed data. Values represent mean ± SD of 3 independent experiments, n = 15, p-values for EB1: 0.7824, 0.0014, 0.0044, p-values for α-tubulin: 0.1421, 0.0099, 0.0042. (**b**) Graphic showing how astral/spindle MT signal was quantified. (**c**) Metaphase HeLa Kyoto cells stably expressing GFP-EB3 transfected with control/MISP siRNA and FLAG-IQGAP1 were imaged with a spinning disc microscope every 2 seconds over one minute. Kymographs from 5-μm-thick sections around the spindle poles (red rectangle) show the dynamics of EB3 comets. Length, zenith angle and number of EB3 comets were measured as illustrated. (**d**) Representative kymographs and quantifications of astral MT dynamics. Length (stability) and angle (speed) of 5 EB3 comets per centrosome were averaged and compared with one-way ANOVA with Bonferroni’s tests, n = 16, ****p < 0.0001. (**e**) Single plane confocal images showing p150^glued^ localization in mitotic HeLa cells transfected with control or MISP siRNA or MISP siRNA and GFP-IQGAP1. Equatorial planes with the strongest cortical accumulation of p150^glued^ are shown. Scale bar: 5 μm. Chart: Quantification was carried out using ImageJ by dividing the strongest line intensity of p150^glued^ at the cortex by the intensity of the same line in the cytosol right below the cortex. Values represent mean ± SD of 3 independent experiments, n = 12, p-values: 9,99 × 10^−4^ and 0,0058. WB shows MISP downregulation. Images were gained from the same Western blot membrane.

MISP seems to play a role in spindle orientation via capturing the astral MTs at the cell cortex in mitosis^2^. To strengthen our data suggesting that IQGAP1 acts downstream of MISP we asked whether IQGAP1 is involved in MISP-induced stabilization of astral MTs. We checked if IQGAP1 OE was able to rescue the loss of astral MTs upon MISP KD. To visualize the MT plus ends, we stained for EB1, a MT + TIP binding protein. While MISP depletion led to a reduction of astral EB1 intensity, IQGAP1 OE could compensate for the loss of MISP and restored normal astral/spindle EB1 distribution (Fig. 6a,b). Loss of astral MTs could also be rescued by IQGAP1 OE in cells where MTs were stained with α -tubulin (Fig. 6a,b). To further characterize how MISP regulates the dynamics of astral MTs, we applied short-term live-cell imaging to mitotic HeLa cells stably expressing GFP-EB3^22^. Timely tracking of EB3 comets in control/MISP siRNA treated cells revealed that astral MTs emanating from the centrosomes of MISP-depleted cells both grew slower and were reduced in length (Fig. 6c,d). These results suggest that MISP KD leads to a decreased stability of astral MTs where most of them do not reach the cell cortex. Strikingly, IQGAP1 OE in MISP-depleted cells restored the speed and track-length of these plus-tip comets demonstrating a joint role of MISP and IQGAP1 in stabilization of astral MTs at the cell cortex. Together these data imply that the stabilization of astral MTs by MISP is dependent on IQGAP1.

The dynein/dynactin complex at the cell cortex is crucial for proper spindle positioning^23^. MISP was shown to negatively regulate the cortical distribution of the dynactin subunit p150^glued^ ^2^. Interestingly, Cdc42 depletion led to a similar effect on the localization of p150^glued^ as MISP^12^. Therefore we checked, if IQGAP1 overexpression could rescue the aberrant cortical accumulation of p150^glued^ upon MISP depletion. Indeed we found, that IQGAP1 OE after MISP KD normalized the cellular distribution of p150^glued^ (Fig. 6e). p150^glued^ could therefore be an effector in regulating astral MT stability downstream of MISP and IQGAP1. However, more experiments are required to substantiate this hypothesis. Taken together, our results suggest that IQGAP1 acts as a downstream effector of MISP to stabilize astral MTs for proper spindle orientation.

## Discussion

Orientation of the cell division axis within multicellular organisms has been a major research focus for over a century. So far, little is known about how the proteins regulating this process collaborate to ensure accurate cell division orientation. Our previous data have shown that the actin-binding protein MISP also regulates spindle orientation by affecting the distribution of p150^glued^, a subunit of the dynein-dynactin complex at the cell cortex^2^. However, the interaction is not direct suggesting the presence of other proteins linking p150^glued^ and MISP. In this study we identify IQGAP1 as a binding partner and a downstream effector of MISP in spindle orientation. Depletion of MISP leads to an increase in the accumulation of IQGAP1 at the cell cortex, which is dependent on active Cdc42. Furthermore, we find that IQGAP1 is required for MISP-dependent stabilization of astral MTs and correct localization of p150^glued^ to ensure faithful orientation of the mitotic spindle. Interestingly, we found that Plk1-dependent phosphorylation of MISP^2^ did not influcence either binding or localization of lQGAP1 (Supplementary Figs 1d and 2b).

**Figure 7:**
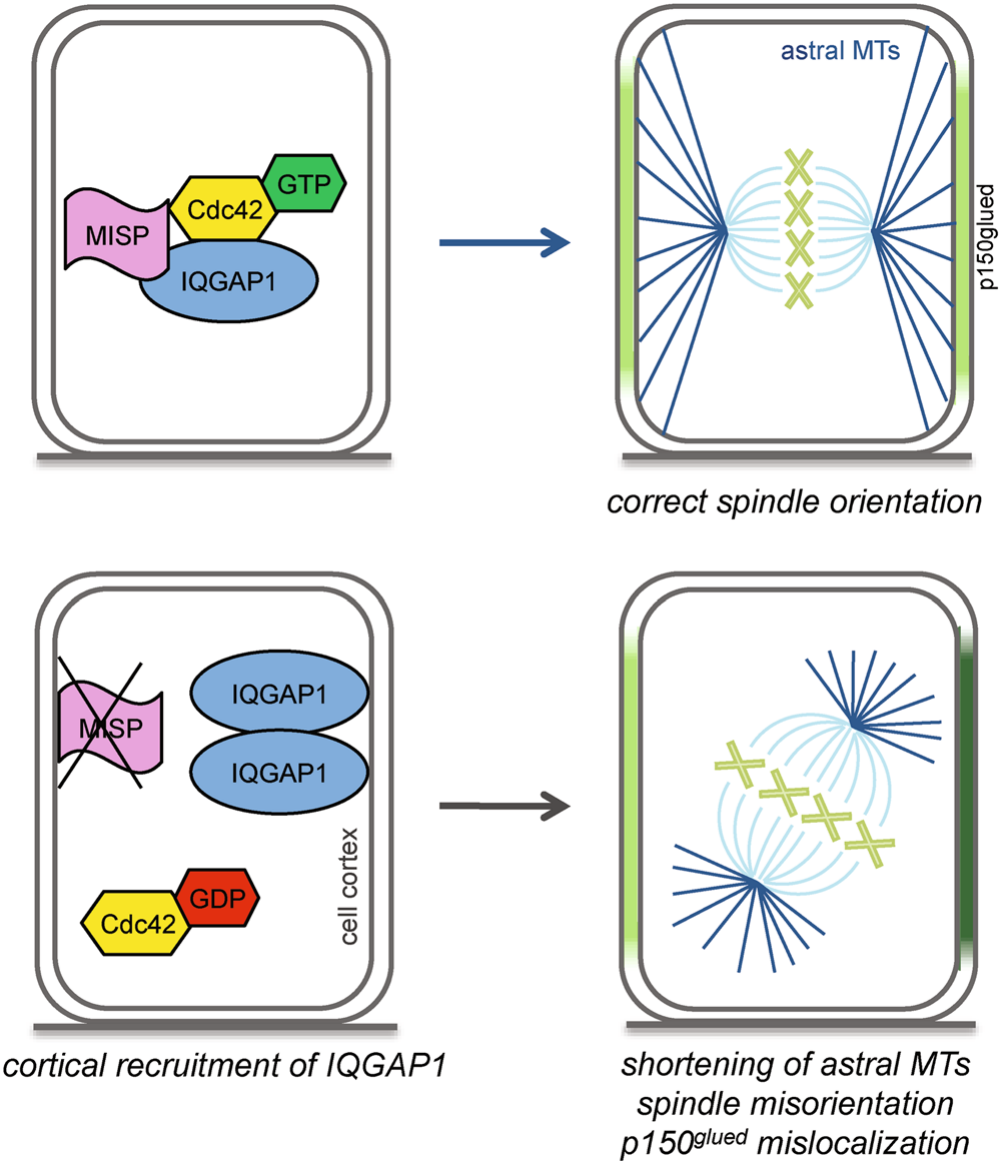
Model summarizing the effects of MISP KD on IQGAP1 and Cdc42 and thereby astral MTs and mitotic spindle orientation. In control cells (upper part) MISP contributes to the stabilization of active Cdc42 by IQGAP1 ensuring proper spindle orientation. Upon MISP depletion (lower part), IQGAP1 is recruited to the cell cortex losing its affinity towards active Cdc42 leading to decreased active Cdc42 levels. At the same time, p150^glued^ is mislocalized at the cortex leading to an asymmetric polarization (shown in dark green). These in turn can induce shortening of astral MTs and spindle misorientation.

Activity of IQGAP1 was supposed to be regulated by a conformational change^21,24^. In the open conformation, IQGAP1 is able to bind Cdc42, while in the closed conformation, when the C-terminus folds on the Cdc42-binding region, binding to Cdc42 is repressed. We hypothesize that MISP binding is necessary for preserving IQGAP1 in its open, active form by binding to its C-terminus (Fig. 1a,d and Supplementary Fig. 1c) and so it enables IQGAP1 to bind and stabilize active Cdc42 (see Fig. 7 for model). Upon loss of MISP p150^glued^ is mislocalized and asymmetrically distributed at the cell cortex. It has been previously shown that dynein is able to capture MT ends, inhibit growth and trigger MT catastrophes, which could lead to a shrinkage of astral MTs^25^. In contrast, upon loss of MISP conformation of IQGAP1 might shift to the closed form, decreasing its ability to bind active Cdc42 (Fig. 4f) and leading to reduced GTP-Cdc42 levels (Fig. 4c). This closed form of IQGAP1 might accumulate at the cell cortex (Fig. 2a), probably by binding to the E-cadherin/β-catenin complex^8,18^. Overexpression of active Cdc42 after MISP KD may open the conformation of IQGAP1 causing its dissociation from the cortex (Fig. 3a) but might shift the balance too much for proper spindle orientation (Supplementary Fig. 3c). Overexpression of IQGAP1 after MISP KD, however, might only moderately increase the proportion of the open conformation leading to the stabilization of the right amount of active Cdc42 (Fig. 5c) that ensures proper anchoring of astral MTs (Fig. 6a–d) and thereby orderly mitotic progression and spindle orientation (Fig. 5a,b).

IQGAP1 and Cdc42 were shown to be required for correct spindle orientation^26^. The main players involved in the regulation of spindle orientation are Gαi, LGN and NuMA forming the evolutionary conserved ternary complex and the force generator dynein-dynactin complex^27^. Importantly, IQGAP1 was shown to regulate mitotic spindle orientation^5^. In this paper we show that IQGAP1 can also regulate the localization of p150^glued^ downstream of MISP (Fig. 6e). Our data suggest that the rescue function of IQGAP1 after MISP depletion is dependent on its Cdc42-stabilizing activity (Fig. 5a), creating a pathway of MISP-IQGAP1-Cdc42 in spindle orientation. We know that the activity of Cdc42 has to be finely tuned in order to ensure proper mitotic progression and spindle orientation. Both deactivation^11,12^ and overactivation (Supplementary Fig. 3c,d) of Cdc42 leads to spindle misorientation. We know that MISP depletion leads to Cdc42 deactivation through IQGAP1 (Fig. 4c,g) and this could lead to spindle misorientation, however, the exact mechanism is still to be clarified. Future work is needed to shed light on how the MISP-IQGAP1-Cdc42 pathway coordinates the ternary complex components to ensure proper spindle orientation.

Apart from positioning meiotic spindles which lack astral MTs (for review see^28^) mitotic spindle orientation is mediated by the interaction of astral MTs with force generators at the cellular cortex. Defects in astral MTs can affect spindle orientation. Our previous data show that depletion of MISP causes destabilization and shortening of astral MTs^2^ resulting in the lack of interaction between the spindle and the force generators. As overexpression of IQGAP1 can rescue destabilization of astral MTs caused by lack of MISP (Fig. 6a–d), we hypothesize that MISP regulates stability of astral MTs through IQGAP1. This could then affect the cortical localization of the IQGAP1 interacting protein NuMA^5^ and therefore the localization of p150^glued^ (Fig. 6e) and influence astral MT capturing at the cortex. Alternatively, IQGAP1 could exert its function on astral MTs via the cytoplasmic linker protein 170 (CLIP-170)^6^ or the tumor suppressor adenomatous polyposis coli (APC)^7^.

Misorientation of the spindle is implicated in a number of diseases including cancer^29^. IQGAP1 is overexpressed in a variety of human cancers (reviewed in^13^ and^30^). Upregulated IQGAP1 levels which lead to an increase in active Cdc42 pools are associated with enhanced tumor proliferation, invasion and angiogenesis^15^, while overexpression of a dominant-negative IQGAP1 reduced GTP-bound Cdc42 and neoplastic transformation of human breast cancer epithelial cells^15^. Our work identifies MISP as a novel binding partner of IQGAP1. Interestingly MISP is also overexpressed in a variety of human cancer cells but not in non-transformed cell types^3^ (Settele and Hoffmann, unpublished). Blocking the formation of the MISP/IQGAP1/Cdc42 complex might have therapeutic potential for specific cancer types. Targeting either the MISP/IQGAP1 or the Cdc42/IQGAP1 interaction may decrease the amount of active Cdc42 in cancer cells and thereby prevent tumor progression.

## Materials and Methods

### Antibodies

The following antibodies were used in this study: anti-MISP (rb)^2^ anti-MISP (ms, kind gift from Alwin Krämer)^3^, anti-IQGAP1 (rb, Abcam 86064), anti-IQGAP1 (ms, Santa Cruz sc-376021), anti-Cdc42 (ms, Cytoskeleton, ACD03), anti-pericentrin (rb, Abcam ab4448), anti-β-actin (ms, Calbiochem JLA20), anti-α-tubulin (ms, Sigma B-5-1-2, T5168), anti-GFP (rb, Novus Biologicals NB600-308), anti-FLAG M2 (ms, Sigma F1804), anti-GST Z5 (rb, Santa Cruz sc-459), anti-EB1 (ms, BD Biosciences 610535), anti-pAkt (S473, ms, CST #587F11), anti-Akt (rb, CST #9272), anti-p150^glued^ (ms, BD Biosciences 612709). Secondary antibodies: Alexa 488/568/647-coupled anti-rabbit/mouse IgG (Molecular probes).

### Mammalian cell culture and transfections

Cells were cultured in Dulbecco’s Modified Eagle’s Medium (DMEM, Sigma) with 1 g/l glucose for HeLa, HeLa Kyoto and A549 cells (ATCC) or 4.5 g/l glucose for HEK293T and MCF-7 cells (ATCC) supplemented with 10% FBS (Lonza) and 1% pen/strep (Sigma). Cells were split at 80–90% confluence. Cell line authentication (based on SNP-profiling) was performed by Multiplexion, Heidelberg. Mycoplasma tests were conducted on a monthly basis (LookOut Mycoplasma PCR Detection Kit, MP0035-1KT, Sigma). The IQGAP1 KO cell line was established as reported before in^20^. The HeLa Kyoto cell line stably expressing GFP-EB3 was a kind gift from Jan Ellenberg, EMBL^22^.

Cells were transfected with the plasmid constructs using polyethylenimine (PEI, Polysciences) at a final concentration of 5 µg/ml in serum-free DMEM the day after seeding. Medium was changed to serum-supplemented DMEM after 4 hours. Cells were harvested 20–24 hours post-transfection.

Transfection of cells with siRNA (40 nM final concentration) was performed using reverse transfection method with Lipofectamine® 2000 (Invitrogen), according to the manufacturer’s instructions. Medium was replaced with DMEM after 4–20 hours. Cells were harvested 48 h after siRNA transfection. Depletion efficiency was always controlled by Western blotting.

### Complex Immunoprecipitation

For analysis of ternary protein complexes, sequential IPs were performed. First, the FLAG-tagged protein was immunoprecipitated from the cell lysates with FLAG M2 beads for 2 hours, then washed and eluted for 30 min on ice with 500 ng/µl 3xFLAG peptide in 100 µl lysis buffer. 10 µl of the eluate was spared for later analysis. 400 µl lysis buffer and 20 µl GFP trap beads were added to the eluate and incubated for 2 h at 4 °C. After a triple wash, bound proteins were eluted by 5-min boiling in 2 × Laemmli buffer.

### Protein purification and pull-down assays

All recombinant proteins were expressed in *E*. *coli* BL21-Rosetta. Protein expression was induced overnight at 18 °C. Bacterial pellets were resuspended in *E*. *coli* lysis buffer (50 mM Tris-HCl, pH 7.5, 250 mM NaCl, 1 mM MgCl_2_, 5% glycerol, 1 mM DTT, 10 µg/ml TPCK, 5 µg/ml TLCK, 2 mg/ml aprotonin, 2 mg/ml leupeptin, 20 mg/ml trypsin inhibitor) and proteins were extracted by sonication and lysates were cleared by centrifugation. GST-IQGAP1, GST-His-Cdc42 and MBP-MISP were natively purified by single-step affinity chromatography using glutathione agarose CL-4B beads (Sigma-Aldrich) or amylose beads (NEB) according to the manufacturer’s instructions.

To analyze direct interactions, GST/MBP pull down assays were performed. 15 µg MBP-MISP was incubated with 15 µg GST-tagged protein or equimolar amount of GST/MBP alone as control in 500 µl lysis buffer for 30 min at 4 °C. Thereafter, 10–10 µl settled glutathione agarose/amylose beads were added and the mixtures were incubated for 1–2 h rotating at 4 °C. After a triple washing step with lysis buffer, bound proteins were eluted by boiling in 2 × Laemmli buffer for 5 min. Samples and 2% inputs were analyzed by SDS-PAGE and western blotting.

### Immunofluorescence Microscopy

Cells grown on coverslips were fixed in 4% paraformaldehyde (PFA) for 5–10 min at room temperature (or in methanol for 5 min at −20 °C for α-tubulin, p150^glued^ and EB1 staining). After washing with PBS, cells were permeabilized and blocked with IF solution (3% BSA, 0.5% Triton X-100 and 0.02% sodium azide in PBS) for 30 min at RT. Samples were incubated for 1–2 hours with primary antibodies diluted in IF solution. After a triple wash with IF solution, coverslips were incubated for 30–60 min with the appropriate Alexa 488/568/647-coupled secondary antibodies (Molecular Probes) diluted in IF solution. Then, for staining nuclei, coverslips were kept in 1 µg/ml Hoechst 33258 solution (in PBS) for 5 min followed by a 5-min PBS wash. Coverslips were dried and mounted onto glass slides with Mowiol mounting medium. Samples were analyzed either with Zeiss ObserverZ1 inverted microscope or with a Zeiss LSM-700 confocal microscope with the following settings: Zeiss motorized inverted Observer.Z1 equipped with a mercury arc burner HXP 120 C and a live-cell chamber for temperature, CO_2_ and humidity control. Illumination: 365/470/555/590, detection: gray scale CCD camera AxioCamMRm system and a 63×/1.4 Oil Pln Apo DICII objective. A Zeiss Apotome optical sectioning device with structured illumination was used during z-stack imaging for near-confocal images. Zeiss LSM-700: Upright motorized Zeiss Imager.Z2 with a 63×/1.4 Oil DIC III objective. Laser lines: 405/488/555/639 nm.

Z-stacks were taken at an interval of 0.5 µm and with a 0.05 µm resolution. Stainings for samples to be compared were done in parallel and images were captured under the same exposure conditions. Images were processed and analyzed with ImageJ software^31^. Single planes or sum projections were used for quantifications, maximum z-stack projections for visualization purposes.

### Statistics

Experiments were repeated at least three times. Unless indicated otherwise, statistics were performed from the mean values using unpaired, two-tailed t-tests with 95% confidence interval in the Prism Software (Graphpad). Multiple comparisons were conducted with one-way ANOVA analysis using Bonferroni’s multiple comparisons test. No data point was excluded. Most of the graphs were generated with Microsoft Excel for illustration purposes; data analysis was done with Prism. Data are presented as mean ± SD. On the graphs, p-values are marked as follows: ****p < 0.0001, ***p < 0.001, **p < 0.01, *p < 0.05, ns – not significant (p > 0.05).

### Data availability

All the relevant data are in the article and its supplementary files or available from the authors upon request.

## Electronic supplementary material

Video 1

Video 2

Video 3

Supplemental information
