# Supplementary material for: MISP regulates the IQGAP1/Cdc42 complex to collectively orchestrate spindle orientation and mitotic progression
Source: Sci Rep. 2018 Apr 20;8:6330. doi: 10.1038/s41598-018-24682-8 (PMC5910412; doi:10.1038/s41598-018-24682-8)
Supplement: Supplementary file 4 — Supplemental information [file 41598_2018_24682_MOESM4_ESM.pdf]

MISP regulates the IQGAP1/Cdc42 complex to collectively orchestrate spindle orientation and mitotic progression

Barbara Vodicska[1], Berati Cerikan[2], Elmar Schiebel[2] and Ingrid Hoffmann[1,*]

[1] *Cell Cycle Control and Carcinogenesis, F045, German Cancer Research Center, DKFZ, 69120 Heidelberg, Germany.*

[2] *Zentrum für Molekulare Biologie der Universität Heidelberg (ZMBH), DKFZ – ZMBH Alliance, Im Neuenheimer Feld 282, 69120 Heidelberg, Germany*

* *Correspondence: Ingrid.Hoffmann@dkfz.de*

SUPPLEMENTARY INFORMATION

Supplementary Figures

Supplementary Figure Legends

Supplementary Experimental Procedures

Supplementary Video Legends

Supplementary Figure 1.

a

Hit nr	Accession	Description	Score	Mass (Da)	Match es	M. sig.	Seqs	Seqs sig.	Coverage (%)
15	CDK1_HUMAN	Cyclin-dependent kinase 1 OS=Homo sapiens GN=CDK1 PE=1 SV=3	593	34131	26	25	10	10	41.8
72	IQGA1_HUMAN	Ras GTPase-activating-like protein IQGAP1 OS=Homo sapiens GN=IQGAP1 PE=1 SV=1	109	189761	3	2	3	2	2.4
82	CDC42_HUMAN	Cell division control protein 42 homolog OS=Homo sapiens GN=CDC42 PE=1 SV=2	95	21587	5	3	3	2	25.1
119	MARE1_HUMAN	MT-associated protein RP/EB family member 1 OS=Homo sapiens GN=MAPRE1 PE=1 SV=3	68	30151	2	1	2	1	11.2

b

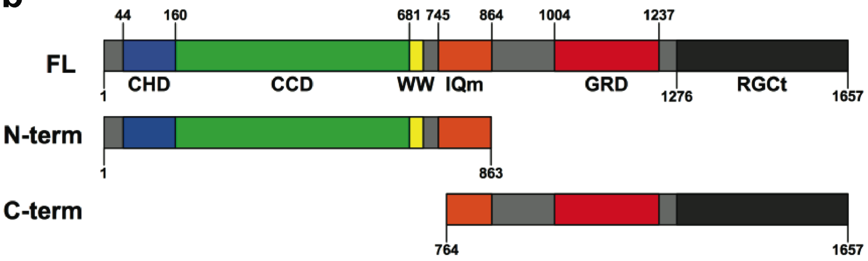

c

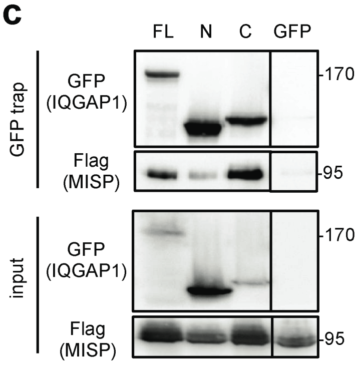

d

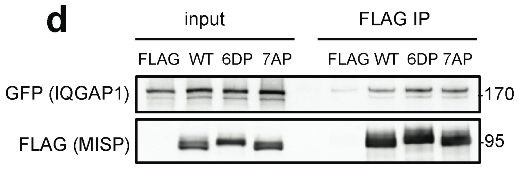

Supplementary Figure 2.

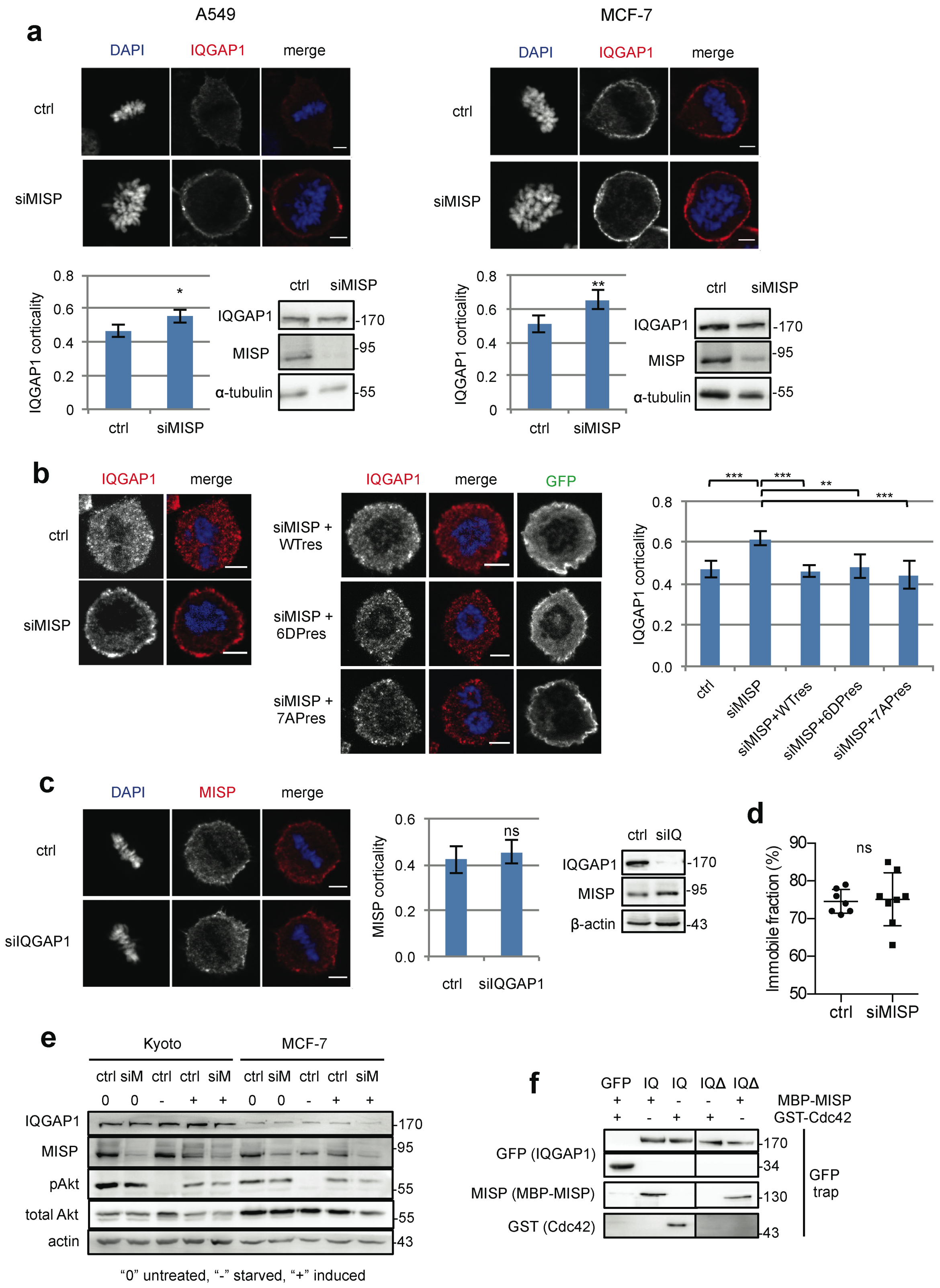

Supplementary Figure 3.

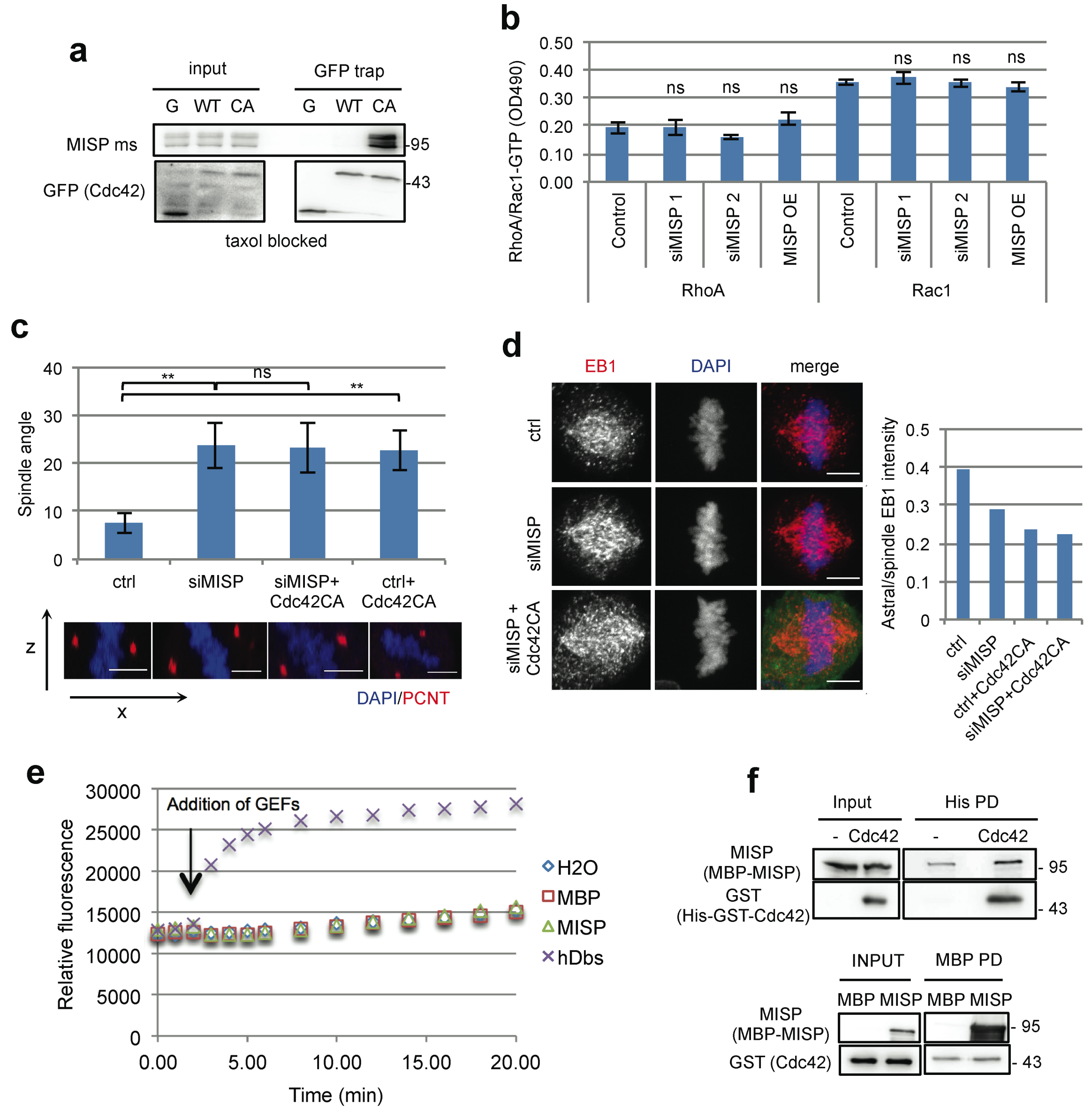

Supplementary Figure 4.

Uncropped blots to Fig. 1.

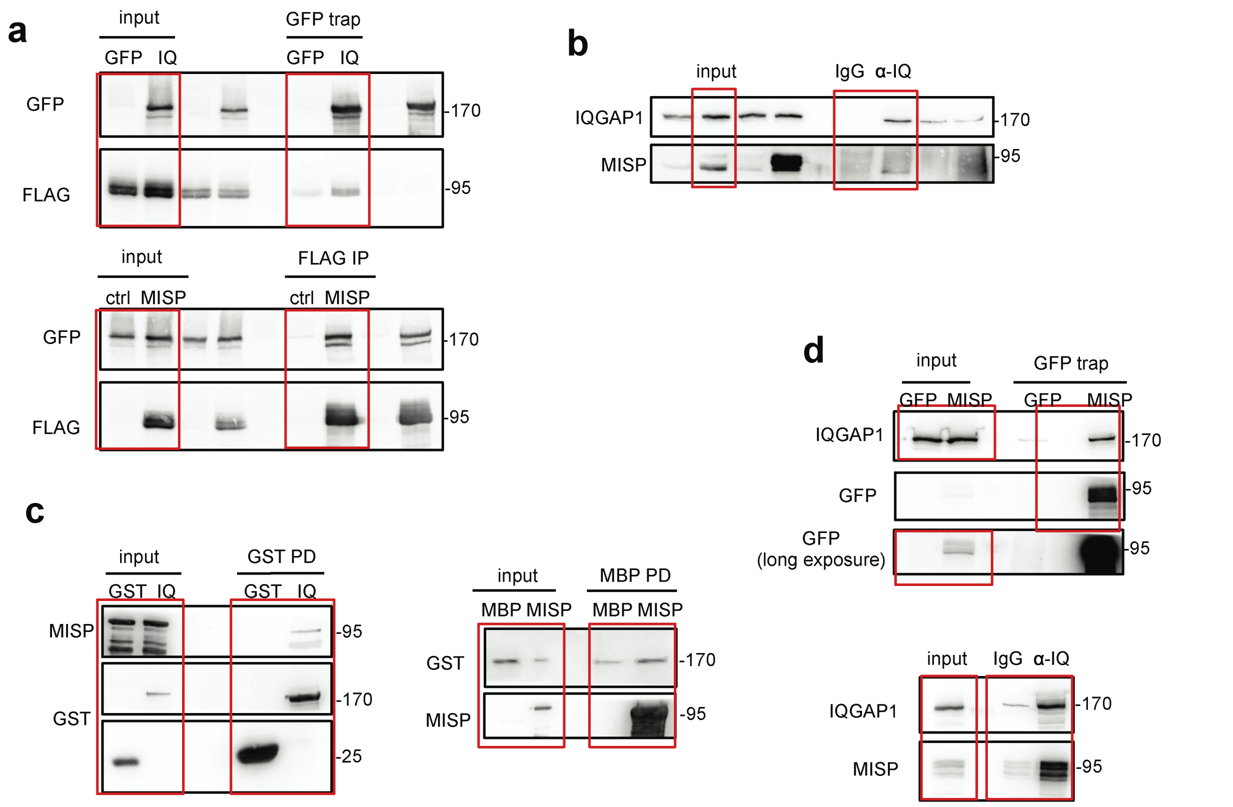

Uncropped blots to Fig. 4.

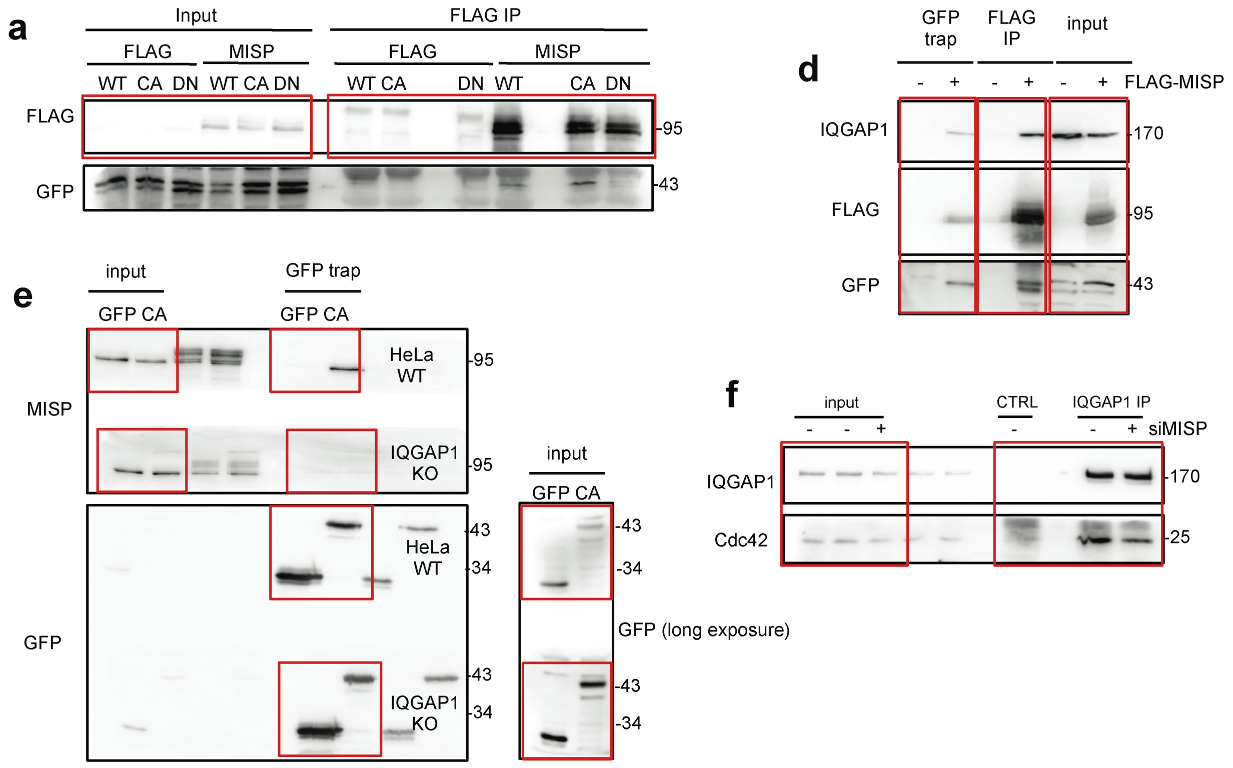

Supplementary Figure 4. continued

Uncropped blots to Supplementary Fig. 1.

c

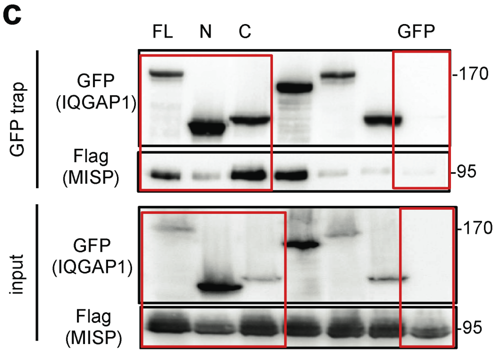

Uncropped blots to Supplementary Fig. 2.

f

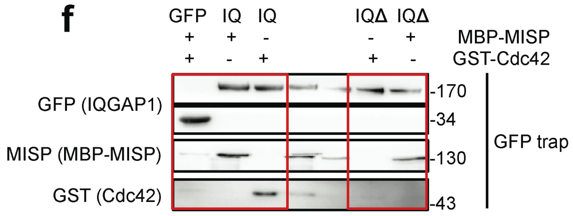

Uncropped blots to Supplementary Fig. 3.

a

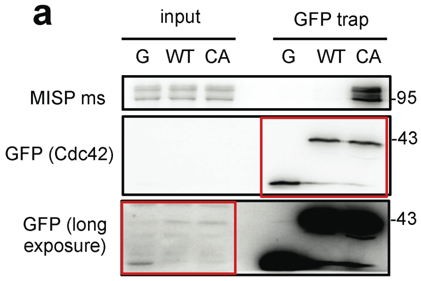

f

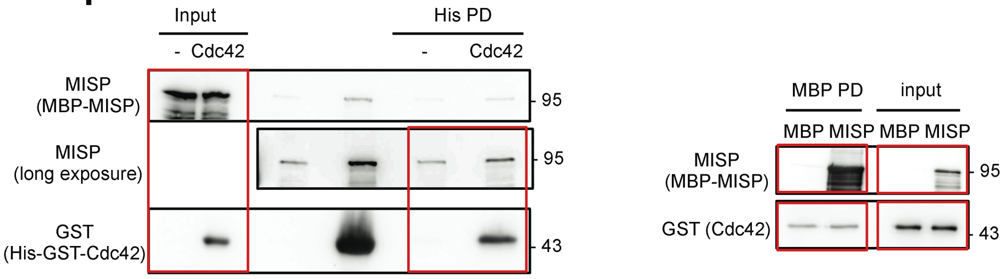

Supplementary Figure Legends

Supplementary figure 1. MISP interacts with IQGAP1. (**a**) HeLa cells were transfected with FLAG or FLAG-MISP. Cell lysates were subjected to immunoprecipitation with FLAG M2 beads and co-precipitating proteins were analyzed using mass spectrometry. The database search was performed with Mascot. 256 specific interaction partners were identified. Hit nr – protein ranking based on score, Accession – accession number of the identified protein, Score – summed petide scores, Mass – protein mass in Dalton, Matches – number of peptides matched, M. sig. – significant matches, Seqs (sequences) – number of unique peptides, Seqs sig. – significant sequences, Coverage – percentage of sequence coverage identified. Proteins relevant for this study are shown. (**b**) Schematic representation of the domain structure and truncation constructs of IQGAP1. (**c**) FLAG-MISP was co-expressed with full-length (FL), N-terminal (N) or C-terminal part (C) of GFP-tagged IQGAP1 or GFP alone in HEK293T cells. GFP trap experiment shows co-precipitation of FLAG-MISP with the different IQGAP1 constructs. (**d**) FLAG empty vector (FLAG) FLAG-MISP wild-type (WT), Plk1 phospho-mimicking (6DP) and phospho-deficient (7AP) mutants were co-expressed with GFP-tagged IQGAP1 in HEK293T cells. The cell lysates were subjected to FLAG immunoprecipitation and co-precipitating IQGAP1 was analyzed by WB.

Supplementary figure 2. Loss of MISP leads to cortical accumulation of IQGAP1. (**a**) Fluorescent images show the localization of endogenous IQGAP1 in mitotic control and MISP KD A549 (left) and MCF-7 (right) cells. Single equatorial images, scale bar: 5 µm. Charts: Cortical accumulation of endogenous IQGAP1 was quantified as in Fig. 2b. Values represent mean ± SD of 3 independent experiments, n=15. Western blots show knockdown efficiency. (**b**) Cortical localization of IQGAP1 in control, MISP KD, MISP KD and MISP mutants overexpressing, mitotically arrested (nocodazole) HeLa Kyoto cells was visualized and quantified as in Fig. 2b. WT - wild-type, 6DP - Plk1 phospho-mimicking and 7AP - Plk1 phospho-deficient mutant. Single equatorial images, scale bar: 5 µm. (**c**) Cortical localization of MISP in control and IQGAP1-depleted mitotic HeLa Kyoto cells was visualized and quantified as in Fig. 2b. Values represent mean ± SD of 3 independent experiments,

n=15. WB shows KD efficiency. (**d**) Quantification of the immobile fraction of the FRAP experiment shown in Figure 2d-e, p=0.8513. (**e**) Immunoblot of control and MISP KD samples for Akt activation in untreated, serum starved (20 h) and induced (30 min 20% FBS after 20 h starvation) conditions in HeLa Kyoto and MCF-7 cells. (**f**) Half-in-vitro experiment shows the binding of MISP and Cdc42 to IQGAP1 (IQ) and IQGAP1ΔCdc42 (IQΔ). GFP/GFP-IQGAP1/GFP-IQGAP1ΔCdc42 was pulled down from HEK293T cell lysates, washed and incubated with recombinant MBP-MISP or GST-Cdc42.

Supplementary figure 3. MISP interacts with Cdc42. (**a**) IP experiment of taxol blocked HeLa cells transfected with GFP or GFP-Cdc42 WT/CA. Co-precipitating MISP was detected in the eluate. (**b**) HeLa Kyoto cells were transfected with control or MISP siRNAs or a GFP-MISP plasmid. Active RhoA and Rac1 levels were quantified using the Rho G-LISA activation assay from Cytoskeleton. Values represent mean ± SD of 3 independent experiments, one-way ANOVA with Bonferroni's test. (**c**) Spindle angle relative to the substratum was measured in mitotic HeLa cells inducibly expressing GFP-Cdc42CA treated with control or MISP siRNA in the presence or absence of doxycycline. Data represents mean ± SD of 3 independent experiments, n=15, one-way ANOVA with Bonferroni's. Lower panel: representative x-z side views. Centrosomes were visualized by pericentrin (PCNT) staining, scale bar: 5 µm. (**d**) HeLa cells inducibly overexpressing Cdc42CA were treated with control or MISP siRNA in the presence or absence of doxycycline and immunostained for the plus-tip binding protein EB1. Maximum projection of z-stack images were used for visualization, scale bar: 5 µm. Chart: astral/spindle EB1 intensity was quantified in sum projections as in Fig. 6b, n=20. (**e**) In vitro Cdc42-GEF assay was performed with purified MBP-MISP, MBP alone, water as a negative and hDbs – a proven Cdc42 GEF – as a positive control. Fluorescence intensity, which is proportional with the amount of produced GTP-Cdc42, was measured over time after the addition of the purified proteins. (RhoGEF exchange assay, Cytoskeleton). (**f**) In vitro interaction between purified MBP-MISP and GST-His-Cdc42 was studied by His and MBP pull-down experiments.

Supplementary figure 4. Uncropped blots.

Supplementary Experimental Procedures

Plasmid constructs

Plasmids: pEGFP-IQGAP1 (Addgene #30112), pcDNA3-EGFP-Cdc42-WT / CA(Q61L) / DN(T17N) (Addgene #12975, #12986, #12976, respectively), pEGFP-IQGAP1-N (1-863) and pEGFP-IQGAP1-C (764-1657) were kind gifts from Fernando Martin-Belmonte, pEGFP-IQGAP1ΔCdc42 (d1054-77)[1]: two-PCR fragment ligation from pEGFP-IQGAP1 and cloned back into pEGFP-IQGAP1 with BsmBI sites, GST-IQGAP1: IQGAP1 was PCR amplified and cloned into the pGEX-4T-3 vector with XmaI and XhoI, pEGFP-MISP and FLAG-MISP (pCMV-3Tag-1-MISP) WT, 6DP and 7AP were cloned as described before [2], cloning of the pcDNA5/FRT/TO_GFP-IQGAP1 plasmid was carried out using the IN-FUSION cloning technique from pEGFP-IQGAP1 into the pcDNA5/FRT/TO vector, pFAT2_His(6)-GST-Cdc42 was created as described in [3]. The 6xHis-FLAG-IQGAP1 construct was created from pEGFP-IQGAP1 using NEB Gibson Assembly Cloning Kit (NEB #E5510). The identity of all constructs was verified by sequencing (LGC Genomics).

siRNAs

siRNAs were ordered from Eurofins: control siRNA (firefly luciferase, GL2): 5'-AACGUACGCGGAAUACUUCGAdTdT-3', siMISP: 5'-GUGUCCAAGUUGU GGAUGAdTdT-3', siMISP-2: 5'-ACUCGGUGUCUGAGUCUCCCUUCUU-3', siIQGAP1: 5'-TGCCATGGATGAGATTGGAdTdT-3'.

Generation of HeLa cells inducibly expressing GFP-IQGAP1 or GFP-Cdc42CA

GFP-IQGAP1 and GFP-Cdc42CA were PCR amplified from pEGFP-IQGAP1 and pcDNA3-EGFP-Cdc42-Q61L (Addgene #30112 and #12986) and cloned into a pcDNA5/FRT/TO vector (Invitrogen). The cloned plasmids were then transfected into Flp-In TRex tetracycline transactivator HeLa cells together with the Flp recombinase encoding plasmid pOG44 (Invitrogen). Hygromycin-resistant colonies were pooled and expanded. Transgene expression was induced with 1 µg/ml doxycycline (SigmaD9891) overnight.

Preparation of cell lysates

Cells were harvested in lysis buffer (150 mM Tris-HCl, pH 8.5, 200 mM NaCl, 0.5% Triton X-100, 0.1% NP-40, 10 mM ß-glycerophosphate, 5 mM NaF, 2 mg/ml aprotonin, 2 mg/ml leupeptin, 20 mg/ml trypsin inhibitor, 20 µg/ml TPCK, 10 µg/ml TLCK, 1 mM Na_3VO_4). After 30 min incubation on ice, lysates were cleared by centrifugation at 16 000 rcf for 7 min. Protein concentration was determined based on the Bradford method (Bio-Rad Protein Assay).

Immunoprecipitations (IPs)

For IPs with overexpressed proteins, cells from one 15-cm dish were used. FLAG IPs were performed using FLAG M2 affinity beads (Sigma). For GFP IPs, home-made GFP trap beads were used. Briefly, GFP-binding protein (#49172, Addgene) was purified from *E.coli,* and after size-exclusion chromatography, covalently coupled to Sepharose beads [4]. Cells for mitotic interactions were pretreated with 250 ng/ml nocodazole (AppliChem) or 100 nM taxol (Sigma) for 17 h. For endogenous IPs, lysates from 10 cm dishes were precleared with sepharose CL-4B beads (Pharmacia Biotech) on a rotating wheel at 4°C for 30 min. Precleared cell lysates were first incubated with 2 µg antibodies against the protein of interest (or normal mouse or rabbit IgG as control, Santa Cruz, sc-2025/sc-2027) on a rotating wheel at 4°C. After one hour, 20 µl protein G- (for mouse antibodies) or protein A- (for rabbit antibodies) coupled sepharose beads were added to the reactions and incubated for another hour. Protein complexes were collected by centrifugation and washed three times with lysis buffer. Immunoprecipitated proteins were eluted from the beads by 5-min boiling in 2x Laemmli buffer.

GTPase activation assay

To determine the activation status of the Rho family GTPases, the RhoA/Rac1/Cdc42 G-LISA Activation Assay (BK135) from Cytoskeleton was used according to the kit manual. Briefly, cells were scraped and lysed on ice with lysis buffer and protease inhibitors provided with the kit, supplied with phosphatase inhibitor cocktail (Roche, 04906845001). After clearing the lysate by centrifugation (10,000 g), protein concentrations were measured and

equalized. GTPase activity measurements were carried out according to the manufacturer's protocol.

Cdc42 GEF assay

To find out if MISP is a guanine-nucleotide exchange factor for Cdc42, the RhoGEF exchange assay (BK100) from Cytoskeleton was used according to the manufacturer's instructions. Briefly, purified Cdc42 was incubated with mant-(N-methylanthraniloyl)-GTP, a fluorophore-labeled GTP analog, whose intensity increases dramatically when bound to the GTPase. Fluorescence intensity is measured over time after the addition of the purified potential GEF (MISP) in different concentrations or the positive control Dbs (a proven Cdc42 GEF) with a plate reader at 440 nm (excitation 360 nm).

Quantification of cortical IQGAP1 signals

For quantification of cortical IQGAP1 intensity, cells were fixed with PFA and stained with the rabbit IQGAP1 antibody (Abcam). In case of mitotic cells, the equatorial z-section was used to calculate the relative intensity of the cortical and overall IQGAP1 signal. A circle was drawn just around the mitotic cell (a) and another one right below the cortex (b). Results were obtained with the integrated densities of the circles using the following formula: (a-b)/a.

Quantification of astral microtubule intensity

Quantifications of astral MT intensities were done on methanol fixed samples stained for α-tubulin or EB1. In sum projection of z-stack images, integrated densities were measured using the oval function of ImageJ of the whole mitotic cell (a) and the mitotic spindle itself (b). Astral/spindle MT intensity was calculated with the following formula: (a-b)/b.

Spindle orientation experiments

Cells were seeded onto glass coverslips freshly pre-incubated with 10 µg/ml fibronectin solution for 30 min at RT. After treatment and staining with pericentrin antibody, z-stack confocal images (0.5 µm/stack) were acquired of metaphase cells with the Zeiss LSM-700 or LSM-710 system. After Z-

projection of a line going through the two centrosomes in x-y dimension, the angle of the line connecting the two centrosomes in x-z dimension was measured with ImageJ.

Live cell imaging

The length of mitosis was studied in HeLa cells with a Zeiss Observer Z1 inverted microscope equipped with a living cell chamber. HeLa cells inducibly expressing GFP-IQGAP1 were seeded on a 6-well plate and transfected with control or MISP siRNA. Expression of IQGAP1 was induced with 1 µg/ml doxycycline (SigmaD9891) 8 h before imaging. Cells were imaged 32 h post-transfection for 16 h. During imaging cells were kept at 37°C with 5% CO_2 in a humidified atmosphere. Brightfield images were taken with the 10x objective (0.3 EC PlnN Ph1 DICI) every 3 minutes. The length of mitosis was measured with ImageJ from NEB to anaphase onset on at least 100 cells.

Live imaging of microtubule dynamics

HeLa cells stably expressing EGFP-EB3 [5] were seeded in the wells of a 6-well plate and reverse transfected with control or MISP siRNA using Lipofectamine 2000. The next day cells were transfected with mock or FLAG-IQGAP1 constructs and transferred into 4-well glass-bottom ibidi chambers (ibidi GmbH, Germany). 48 h post-transfection and 1 h after addition of SiR-DNA (SC007, Spirochrome) at 1 µM final concentration for DNA staining, mitotic cells were imaged with a PerkinElmer ERS-6 spinning disc confocal microscope equipped with a Nikon Plan Apo λ 100x NA 1.45 oil immersion objective (working distance 0.13 mm) and a Yokagawa CSU-22 confocal scanning unit. An environmental box built around the microscope allowed for temperature-, and CO_2 control. For excitation, 488 and 640 nm laser lines were used, emission was detected with a Hamamatsu C9100-02 EMCCD camera (1000 x 1000 pixel, 8 µm pixel size) with the following filter sets: (center wavelength [nm]/bandwidth [nm]): "Green": 527/55, "Far Red": dual pass filter 705/90. Image acquisition of z-stacks of 5 planes 0.2 µm apart was performed with the PerkinElmer Volocity software every 2 seconds for 1 minute of cells with a nicely aligned metaphase plate (judged by SiR-DNA

staining). Sum projected z-stacks of the spindles were aligned with the spindle axis parallel to the x-axis. Kymographs were created in ImageJ [6] by selecting a region of 5 µm in height (y-axis) between the centrosomes with the function "reslice" and subsequent maximum projection along the y-axis of the original stack. In this kymograph the slope of traces represent the speed along the x-axis.

Mass spectrometry analysis

Mass spectrometry (MS) analysis was performed at the DKFZ MS Core Facility. For identification of MISP-interacting proteins, FLAG-MISP immunoprecipitates were prepared and resolved by SDS-PAGE. After colloidal blue staining, gel lanes were cut into slices, digested with trypsin after reduction and alkylation of cystines. Tryptic peptides were analyzed by nano LC-ESI-MS/MS using a nano Acquity UPLC system (Waters GmbH, Eschborn, Germany) coupled online to an LTQ Orbitrap XL mass spectrometer (Thermo Scientific). Data were acquired by scan cycles of one FTMS scan with a resolution of 60000 at m/z 400 and a range from 300 to 2000 m/z in parallel with six MS/MS scans in the ion trap of the most abundant precursor ions. Instrument control, data acquisition and peak integration were performed using the Xcalibur software 2.1 (Thermo Scientific, Bremen, Germany).

Database searches were performed against the SwissProt database with taxonomy "human" using the MASCOT search engine (Matrix Science, London, UK; version 2.2.2). MS/MS files from the individual gel slices of each lane were merged into a single search. Peptide mass tolerance for database searches was set to 5 ppm and fragment mass tolerance was set to 0.4 Da. Carbamidomethylation of cysteine was set as fixed modification. Variable modifications included oxidation of methionine and deamidation of asparagine and glutamine. One missed cleavage site in case of incomplete trypsin hydrolysis was allowed. A hit was considered significant if the protein score was above the MASCOT identity threshold. Threshold means that an event would be expected to occur at random with a frequency of less than 1% ($p<0.01$). Proteins were considered as identified if at least one unique peptide

had an individual ion score exceeding the identity threshold. The protein score was ranking criteria (the protein with the highest protein score was ranked first), calculated as the sum of all individual peptide scores. Every peptide got its individual ion score based on probability based scoring, which were summed up to give the protein score. If a peptide was fragmented more than once only the highest peptide score was taken for the protein score. Accession – accession number of the identified protein. Mass – protein mass in Dalton. Matches – number peptides matched. Sequences - number of unique peptides. Coverage – percentage of sequence coverage identified.

Fluorescence Recovery After Photobleaching (FRAP)

HeLa cells inducibly expressing EGFP-IQGAP1 were seeded on a 6-well plate and reverse transfected with control or MISP siRNA using Lipofectamine 2000. The next day cells were transferred into 4-well glass-bottom ibidi chambers (ibidi GmbH, Germany) and IQGAP1 expression was induced with doxycycline. 48 h post-transfection and 1 h after addition of SiR-DNA (SC007, Spirochrome) at 1 µM final concentration for DNA staining. Mitotic cells were imaged on a Leica TCS SP5II confocal microscope with a Leica PL APO 63x/1.4 oil objective. Cells were kept at 37°C with 5% CO_2. For GFP excitation, a 488 nm Argon laser line was used and fluorescence emission was collected between 500 to 560 nm. Mitotic cells with a nicely aligned metaphase plate were imaged, judged by SiR-DNA staining.

For acquiring pre-bleach intensities, five consecutive images were taken at 10% laser power. Then a 4 x 2 µm rectangle at the cell cortex was bleached with 5 laser pulses of 4 lasers (405/458/476/488) at 100% power, each lasting for 1.3 seconds. For recovery measurements 20 single section images were collected at 3s intervals with 10% laser power. Intensities were normalized to pre-bleach data and plots were generated with the FRAP wizard of the Leica LAS AF software. Data points were exported to excel for better visualization.

SUPPLEMENTARY REFERENCES

1 Mataraza, J. M., Briggs, M. W., Li, Z., Frank, R. & Sacks, D. B. Identification and characterization of the Cdc42-binding site of IQGAP1. *Biochemical and biophysical research communications* **305**, 315-321 (2003).
2 Zhu, M. *et al.* MISP is a novel Plk1 substrate required for proper spindle orientation and mitotic progression. *J Cell Biol* **200**, 773-787, doi:10.1083/jcb.201207050 (2013).
3 Bastos, R.N., Penate, X., Bates, M., Hammond, D. & Barr, F.A. CYK4 inhibits Rac1-dependent PAK1 and ARHGEF7 effector pathways during cytokinesis. *The Journal of cell biology* **198**, 865-880 (2012).
4 Kubala, M.H., Kovtun, O., Alexandrov, K. & Collins, B.M. Structural and thermodynamic analysis of the GFP:GFP-nanobody complex. *Protein Sci* **19**, 2389-2401 (2010).
5 Sironi, L. et al. Automatic quantification of microtubule dynamics enables RNAi-screening of new mitotic spindle regulators. *Cytoskeleton (Hoboken)* **68**, 266-278 (2011).
6 Schneider, C.A., Rasband, W.S. & Eliceiri, K.W. NIH Image to ImageJ: 25 years of image analysis. *Nat Methods* **9**, 671-675 (2012).

Supplementary Video Legends

Supplementary Video 1. Mitotic progression in control HeLa cells. HeLa cells inducibly expressing GFP-IQGAP1 were transfected with control siRNA and cell cycle progression was analyzed by time-lapse microscopy. Frames were taken every 3 min, scale bar: 5 µm.

Supplementary Video 2. Mitotic progression in MISP-depleted HeLa cells. HeLa cells inducibly expressing GFP-IQGAP1 were transfected with MISP siRNA and cell cycle progression was analyzed by time-lapse microscopy. Frames were taken every 3 min, scale bar: 5 µm.

Supplementary Video 3. Mitotic progression in MISP-depleted and IQGAP1 overexpressing HeLa cells. HeLa cells inducibly expressing GFP-IQGAP1 were transfected with MISP siRNA and IQGAP1 expression was induced with doxycycline. Cell cycle progression was analyzed by time-lapse microscopy. Frames were taken every 3 min, scale bar: 5 µm.
